# Recent progress in polymeric ultrafine fibrous scaffolds for enabling cell infiltration in tissue engineering

**DOI:** 10.1177/08853282251380622

**Published:** 2025-09-14

**Authors:** S. M. Kamrul Hasan, Prosenjit Sen, Habibur Rahman Anik, Md. Redwanul Islam, Mowshumi Roy, Toufique Ahmed, Abu Naser Md Ahsanul Haque

**Affiliations:** 1 Department of Textile Engineering, 551802 National Institute of Textile Engineering and Research, Dhaka, Bangladesh; 2 Department of Fashion and Textiles, School of Design and Social Context, 5376 RMIT University, Brunswick, VIC, Australia; 3 Department of Yarn Engineering, 421971 Bangladesh University of Textiles, Dhaka, Bangladesh; 4 Department of Textiles, Merchandising and Interiors, College of Family and Consumer Sciences, 1355 University of Georgia, Athens, GA, USA; 5 Department of Chemistry and Chemical & Biomedical Engineering, 8518 University of New Haven, West Haven, CT, USA; 6 Department of Apparel Engineering, 421971 Bangladesh University of Textiles, Dhaka, Bangladesh; 7 Department of Fashion Design and Apparel Engineering, National Institute of Textile Engineering and Research, Savar, Dhaka, Bangladesh; 8 Department of Textile Engineering, 130058 Daffodil International University, Dhaka, Bangladesh; 9 Faculty of Science, Engineering and Built Environment, Institute for Frontier Materials, 206228 Deakin University, Geelong, VIC, Australia

**Keywords:** extracellular matrix, electrospinning, phase separation, nanofiber, microfiber, pore size

## Abstract

The structural features of polymer-based tissue engineering scaffolds engineered to support cell adhesion, proliferation, and differentiation have been consistently and assiduously studied over the past few decades. It is now well known that scaffolds composed of polymers with ultrafine fibrous morphologies produced via electrospinning and integrated porosity, can positively influence cell response. The primary objective of most studies in tissue engineering scaffold development is to create a scaffold that emulates the native in vivo-like environment of extracellular matrices (ECMs). Achieving an even distribution of cells throughout the scaffold is critical for exactly mimicking the native extracellular matrix environment. However, inadequate cell infiltration towards the center of the scaffolds has been a common issue in many studies. Only a limited subset of researchers has successfully identified the structural features of scaffolds that facilitate cell penetration and has consequently introduced innovative scaffolds. This study aims to identify the critical structural features of polymeric scaffolds that facilitate cell infiltration and presents novel ultrafine fibrous scaffolds engineered to enhance uniform cellular penetration.

## Graphical abstract

**Graphical Abstract:**
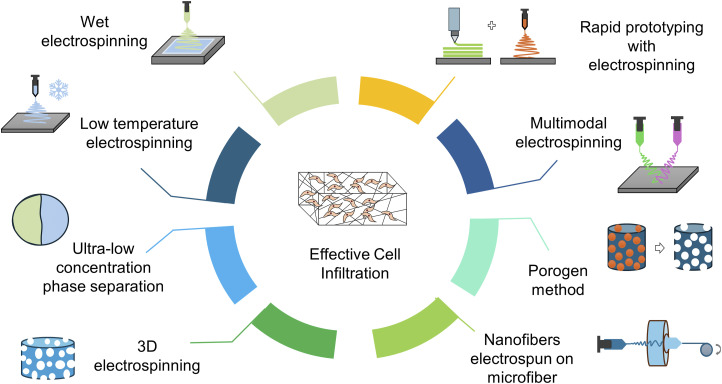

## Introduction

Ultrafine fibrous tissue engineering scaffolds can highly support the treatment of the presence of inflammatory cells in human tissue and organ without other evidence of their inflammatory process. Organ and tissue deficiencies in the cardiovascular blood vessels,^1–4^ neural and neuromuscular,^5^ orthopedic^6,7^ and plastic reconstructive, gastrointestinal, urological, nephrological, and blood transfusion^8^ and skin^9,10^ related human health problems require living tissue regeneration and placement treatment. Tissue engineering works on developing artificial tissues and organs that can replace or augment malfunctioning or damaged ones in the human body.^10–12^

Tissue engineering faces a range of interdisciplinary challenges spanning cell biology, materials science, fabrication, and translational hurdles. Cell sourcing and culture remain critical due to limitations in availability and aging-related functional decline,^13,14^ addressed through organ, explant, and organotypic culture techniques.^15,16^ On the fabrication side, advanced strategies such as electrospinning,^17^ 3D bioprinting,^18^ and decellularization/recellularization^19^ aim to mimic the extracellular matrix, though challenges remain in pore interconnectivity and long processing times.^20,21^ Biomaterial scaffolds must balance biocompatibility, biodegradability, and mechanical integrity, with solutions ranging from polymeric and ceramic scaffolds^22,23^ to hybrid composites and electrospun designs.^24^ Bioreactors, including perfusion and microfluidic systems, help optimize in vitro conditions but remain costly and technically demanding.^25,26^ At the biochemical level, controlled delivery of growth factors such as TGF-β, VEGF, and BMPs is essential but hampered by instability and inconsistent cellular responses.^27–29^ Additional hurdles include biomechanical assessment,^30^ ensuring immunocompatibility,^31,32^ and achieving sufficient vascularization, often pursued via angiogenic factor delivery or microfluidic strategies.^33,34^ Beyond the laboratory, issues of quality control, regulatory compliance, and standardization^35,36^ complicate scale-up and commercialization,^37^ while clinical integration raises patient-specific and long-term viability concerns.^38^ Finally, ethical and legal considerations, including sourcing of hESCs, informed consent, and intellectual property rights, continue to shape the societal acceptance of tissue engineering.^39,40^

An ideal scaffold should hold a number of major characteristics to effectively support tissue regeneration. First, it should have an ultrafine, interconnected three-dimensional porous network that enables cells to attach, migrate, and proliferate throughout the scaffold. Additionally, the scaffold must include proper channels for oxygen and nutrient circulation to ensure cells deep within the scaffold are nourished and to facilitate the removal of cellular waste. Biocompatibility is essential, allowing native tissues or cells to attach and proliferate without adverse reactions. The scaffold should also have the appropriate shape, whether three-dimensional (3D), two-dimensional (2D), or otherwise, depending on the specific application. Finally, it should exhibit suitable biomechanical properties, functionality, and an immunogenicity profile that matches the needs of the tissue it aims to regenerate.^41^

A major human health problem is damaged organ or tissue that originated either from a disease or an injury.^8^ Numerous works have been published related to the regeneration of tissues offering cell growth on flat glass or polystyrene substrate, with the belief that animal physiology can be truly reproduced using a two-dimensional (2D) cellular monolayer.^42^ However, the culture of cells in 2D overlooks many parameters such as cells’ spatial and temporal organization, which are a must to generate a native three-dimensional (3D) extracellular matrix (ECM) like environment. 3D organization of cells can ensure controlled cell functions, i.e. division, through proliferation to differentiation and apoptosis. In this regard, researchers have developed a number of three-dimensional models (i.e. cells seeded within 3D matrices) for a variety of tissues where the culture environment considers the spatial organization of the cells.^43–45^ In 3D models, in addition to spatial control, cellular aggregates require careful exchange of nutrients and gases. But challenges with cell death become apparent when aggregate thicknesses of 1–2 mm occur through a lack of mass transfer, i.e. reduced exchange of nutrients and waste metabolites.^46,47^ Thus, in order to create a growth environment that mimics the native 3D ECMs, cells should be introduced into a porous biocompatible substrate.^48^ Today, this is done by introducing cells into a biocompatible porous 3D scaffold or matrix, which once seeded can mimic the natural ECM where cells adhere, proliferate and migrate. Apart from using either synthetic or natural polymer scaffolds, special attention has now been paid to the composite materials based on the polymer blends for improved cell functioning with adequate mechanical properties.^49–54^ Sometimes, additional cross-linking ingredients such as Genipin are used to improve the mechanical properties of the blends.^55^ Different types of organs or tissues have different shapes and architectures. As Cellular response on substrates is very reliant on the length scale of substrate’s surface features^56^ and scaffolding surfaces, which are directly interacting with cells, should have alike dimensions as the natural ECM,^57^ scaffolds of various structure and morphology are necessary for each of the different types of tissues. In the native 3D ECM environment, the 3D configuration and nanometer-scaled fibrous morphology have been observed to influence cell behavior in several tissues.^58–61^ For this reason, many research works were carried out in designing 3D fibrous scaffolds for TE to mimic the natural 3D ECM. Several different polymeric materials and processing techniques have been proposed to produce 3D scaffolds with Micro- or Nano- or combined Micro-/Nano- hybrid morphology that provide high cell adhesion, proliferation and migration.^62–66^ But one major problem of most of the approaches, which remains less noticed by the researchers so far, is limited to no spreading of cells inside the scaffold.^67^ The key challenge in this respect of tissue engineering is to find an applicable fibrous (as fibers incorporates contact guidance to the cells to orientate and move rapidly along fibers^68^) scaffold with not only an plenty of active groups and good biocompatibility, but also containing appropriate mechanical properties, biodegradability and porous structures with large interconnected pores as large interconnected pores prevent cells from accumulating at the surface of the scaffold by enabling them to diffuse and distribute throughout the entire scaffold.^69^ While existing reviews provide valuable insights into electrospinning techniques and the general application of fibrous scaffolds in tissue engineering, there is a gap in comprehensive analyses focusing specifically on ultrafine fibrous scaffolds and their role in enhancing cell infiltration. This paper aims to fill this gap by representing some original approaches in fibrous scaffold designing, where high cell infiltration so far has been taken under consideration without negotiating critical cell functions. Overall, this review highlights the unique advantages of ultrafine fibers for enhanced cell infiltration, addresses structural limitations in current scaffolds, and integrates insights from material science, bioengineering, and cell biology to develop next-generation ECM-mimicking scaffolds.

## Importance of infiltration and 3D organization of cells in TE scaffolds

**Figure 1.**
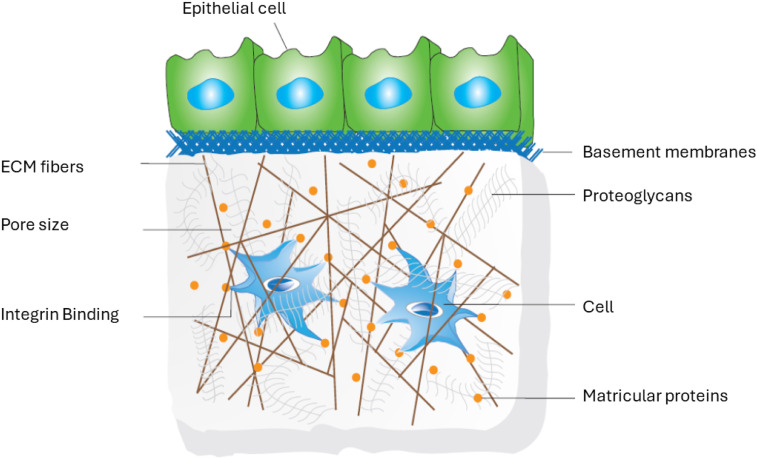
Schematic of ECM 3D environment for engineering cell function.

3D configuration of the matrix affects both the diffusion of solute and binds several effector proteins, such as growth factors and enzymes. Furthermore, the 3D environment is crucial in morphogenetic and remodeling events in 3D ECMs.^70–75^ The 3D organization of cells promote proper transmission of mechanical cues and other receptor expressions between cell-cell and cell-substrate, as in native ECM *in vivo* (Figure 1).

3D organization of cells is also important to achieve the exact shape and structure of their native state when cultured *in vitro*.^48,76^ For example, Proper spatial localization of epidermal growth factor receptor signaling activated by cell secreted autocrine ligands denotes a critical factor in embryonic development and tissue organization as cells use *autocrine loops* (a signaling mode) as sonar system to probe surrounding environment by recapturing the fraction of signals they sent out and thereby helping to define tissue boundary.^77^ The idea of incorporating cells on 3D scaffolds comes mainly because, in 3D environments, cells can grow more branches to receive signals from multiple directions than in 2D environments.^78^ But poor cell infiltration and nutrient transport inside the 3D scaffolds limit their application in tissue engineering, particularly when used for *in vitro* culture environment, because, as time passes, the colonized cells seeded and adhered to the scaffold proliferate and migrate only on the surface, resulting in a necrotic core.^79^ Therefore, an exact mimic of the native 3D environment can only be done *in vitro* when cells can penetrate easily inside the 3D scaffolds from the surface and prevent themselves from flattening.

## Scaffold parameters required for 3D organization of cells

The characteristic requirement of a scaffold (implantable or injectable form) for effective cell functioning *in vivo* or *in vitro* is well known.^80,81^ Along with the fact that cell adhesion to substrate is ‘cell type’ and ‘scaffolding material’ specific,^82^ a nanofibrous scaffold with 3D architecture is well well-defined characteristic for high cell adhesion to the scaffold.^83–86^ Microfiber scaffolds can produce significantly large pores, but several studies showed nanofibrous scaffolds are better for cell adhesion and proliferation than microfiber scaffolds made from the same component due to their (nanofibers) high surface area to volume ratio.^87–93^ Moreover, nano-scale architectures make the cells create more filopodia and contribute to the attachment and proliferation of the cells. But cell migration is limited to only on the seeded surface because of small pore size of 2D nanofibrous scaffold^94,95^ which is not enough for cell infiltration as in many cases, large pore size (several hundreds of micrometer) is necessary for efficient migration of cells throughout the scaffold.^90,96–98^ Even though high porosity and large pore size have positive effect on cell differentiation and matrix production,^99–101^ excessive large pores however might not be possible for the cells to bridge over^90^ and can cause the loss of extracellular matrix proteins into the medium from the scaffold,^102^ hence affecting cell growth negatively.

Another important aspect in this regard is pore interconnectivity and tortuosity (the proportion of the actual path length through connected pores to the shortest linear distance^103^). Interconnected pores allow efficient transfer of nutrients and waste metabolites and permit matrix production.^104^ The interconnected open poor structure can prevent the development of closed-cell morphology and inhomogeneous distribution of cell sizes.^105^ Additionally, a porous surface is known to enhance mechanical interlocking between the implanted scaffolds and the surrounding natural tissue *in vivo*, offering greater mechanical stability at this critical interface^106^ and permit facile invasion of blood vessels for the source of nutrients to the transplanted cells *in vivo*.^107^ So, along with 3D ultrafine fibrous (nano- or micro- scaled fibers) morphology, for high cell infiltration, it is very important to develop scaffolds with high porosity and sufficiently large pores where cells, once adhered to the substrate in efficient culture environment, can proliferate and migrate along the fibers through the inter fiber gaps or pores (well defined macro/micro-pores) towards the inside of the scaffold and organize in 3D.

Therefore, for effective 3D cell organization, scaffolds must combine nanofibrous architecture with high porosity, sufficiently large and interconnected pores, and appropriate surface features to enable deep cell infiltration, proliferation, and migration throughout the structure.

## Fabrication techniques of fibrous scaffolds for high cell infiltration

Although some cultural environments have shown a significant effect on cell infiltration, such as flow perfusion,^108^ but it is only effective for tissue engineering *in vitro* at the cost of a huge number of cells. An effective method to enhance nutrient and cell transfer to the scaffold center, both *in vivo* and *in vitro,* is to model an optimized scaffold with sufficiently large pores. Emphasis has now been given to increasing the pore size of nano-fibrous scaffolds. Several recently published studies show that combining nanofibers and microfibers in a scaffold may increase the pore size of the fibrous construct.^65,92^ Nanofibers then facilitate surfaces for cell attachment and proliferation, whereas microfibers support the structural environment for cell infiltration. Nano-fibrous scaffolds with macro- (>100 µm) or micro-pores can also be created without incorporating microfibers. Here, some of those noble 3D fibrous scaffolds are represented, where the produced scaffolds nearly bear those properties necessary for cell infiltration. To fabricate noble scaffolds that can provide an isotropic arrangement of nano-/micro-scaled environment throughout the scaffold, researchers are now concentrating on the modification of standard fabrication techniques.

### Wet electrospinning

**Figure 2.**
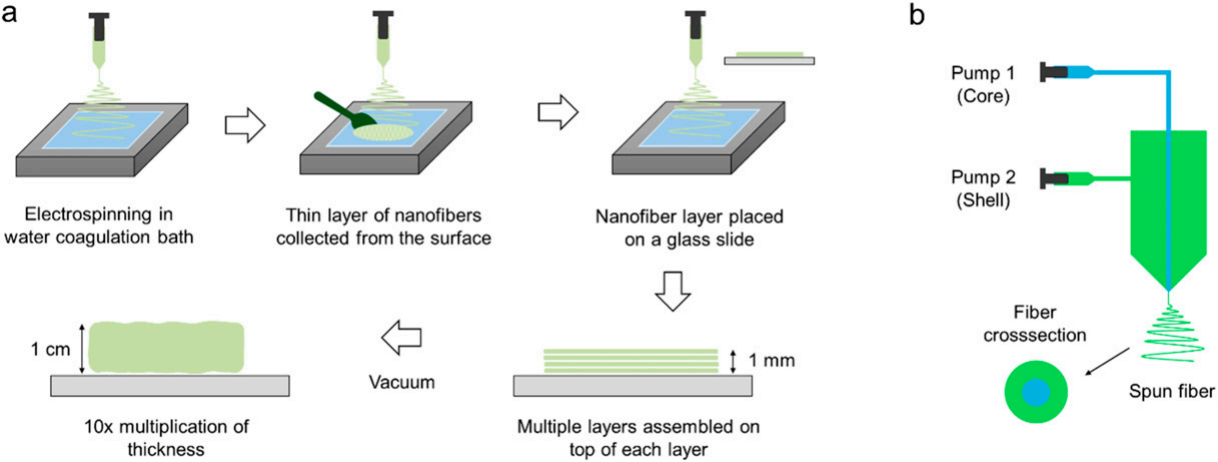
(a) Step by step method of layered hydrospinning through electrospinning and stacking layers of nanofibers followed by vacuuming, redrawn from,^91^ and (b) the concept of core-shell electrospinning.

An effective example of wet electrospinning is ‘Layered hydrospinning’ (Figure 2(a)). 3D poly(*e*-caprolactone) (PCL) nanofibrous scaffold produced by this method has shown a porosity of up to 99% and pores with diameters of over 100 micrometers.^109^ This process is fast, and a more than 1 cm thick scaffold can be produced in a reasonably short time. The difference in the pore size between hydrospun and electrospun nano-fiber scaffolds may be due to water exerting tension on fibers, causing them to widen, resulting in larger pores, evidently due to the high ductility of PCL at room temperature. Although not very significant, large pore size can also be clarified by the fact that, in electrospinning, deposited fibers possess charge that causes the newly deposited fiber to move towards the gap between two adjacent fibers deposited earlier and thus making the inter fiber gap almost half resulting in pores with half the size of original one. However, in hydro spinning, fibers are discharged immediately, and next, falling fibers will not repel towards the center of the pore. The thickness difference exists because of the tendency of layers to separate from each other due to tension exerted by water on the nanofibers while evacuating, as they have a large surface area.^109^ Furthermore, innovative adaptations combining wet electrospinning and coaxial (core–shell) approaches produced 3D porous PCL scaffolds with protein cargo (e.g., BSA), demonstrating sustained release, biocompatibility, and favorable pore architecture conducive to cartilage tissue regeneration.

**Figure 3.**
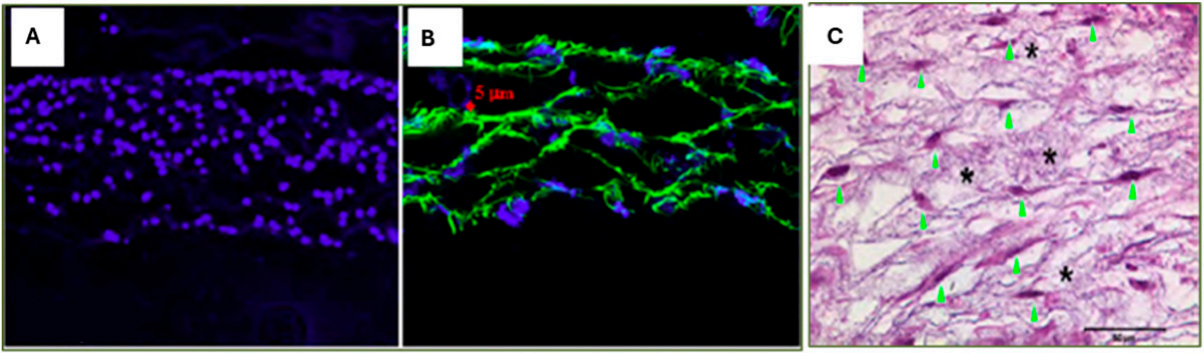
Microscopic images of multilayered cell-fiber constructs: (a) Fluorescent micrograph of DAPI-stained cross-sections of fiber-cell constructs cultured for 2 days. Nuclei = blue. Scale: 200 μm. (b) Confocal microscopic images of cross-sections of formed cell-fiber constructs with a controlled thickness of fiber layer. Scale: 20 μm. Fibers were labelled with FITC (green), and cells were stained blue with DAPI. (c) H&E-stained cross-section of the construct cultured for 7 days. Arrowhead indicates fibroblasts, and asterisks show fibers. Scale: 50 μm. The images shown are representative of three separate experiments. Reprinted from,^111^ Copyright (2023), with permission from Elsevier.

Hydrospinning provides two distinct advantages over typical electrospinning. Those are thicker, porous scaffolds produced in a realistic period and with higher porosity with comparatively large pore size. A similar wet electrospinning method was introduced later by Yang et al., with the exception that no vacuum treatment is necessary.^110^ They developed a layer-by-layer cell/fiber deposited 3D scaffold where cells are seeded on consecutive electrospun layers. They used grounded liquid (including culture medium or Phosphate Buffer Saline, or distilled water) as a fiber collection surface instead of using a solid collector. Single cell types, such as incorporation of osteoblastic cells on PCL/chitosan nanofibers for bone-like tissue formation, or multiple cell type for example incorporating dermal fibroblasts and keratinocytes onto PCL/collagen layers in alternating manner for skin tissue engineering can effectively be used in this ‘L-b-L’ bottom up assembly approach.^110^ Most importantly, cells can be seeded while spinning fibrous meshes. Scaffolds of controlled thickness can be made by fixing the electrospinning time for each layer, and a uniform distribution of cells into the interior of the scaffold (Figure 3) can be obtained by seeding cells on successive layers. A broader review emphasizes the critical role of scaffold porosity and pore size in tissue engineering, showing how specific pore ranges influence cell behavior and tissue formation. Small pores (∼1–2 µm) support skin epidermal attachment, moderate pores (∼2–60 µm) aid dermal migration, cardiovascular, and lung tissue integration, and larger pores (∼40–400 µm) enhance vascularization, nutrient diffusion, and bone tissue regeneration. Optimizing pore size distributions across different tissues is key to improving scaffold functionality and advancing tissue regeneration strategies.^112^ Table 1 summarizes some related methods and key outcomes reported in wet electrospinning.

**Table 1.** Wet-electrospinning methods in tissue engineering and key findings.

Study/technique	Scaffold features	Outcomes	References
Layered hydrospinning	3D PCL nanofibrous scaffold with porosity up to 99% and pore diameters >100 µm	Rapid fabrication of highly porous scaffolds with larger pores	^109^
Layer-by-layer (L-b-L) wet electrospinning depositing PCL/collagen onto culture fluid	Enables simultaneous cell seeding and fiber deposition; thickness control by spinning time	Uniform cell distribution within scaffold	^110^
Wet-electrospun PCL porous 3D scaffolds	Dynamic wet-electrospinning via vortex collection; high porosity (∼99.5%)	Enhanced 3D cell colonization, improved structural complexity for cartilage regeneration	^113^
Core–shell + wet electrospinning	3D tablet-like porous PCL fibers fabricated; optimized pore size and mechanical properties	Sustained protein release for ≥12 days; good porosity and cell infiltration	^114^

Overall, layered hydrospinning enables the rapid fabrication of thick, highly porous 3D PCL scaffolds with large, well-distributed pores, supports cell seeding during scaffold formation, and allows controlled pore architectures that enhance nutrient diffusion, vascularization, and tissue-specific regeneration.

### Core–shell electrospinning

Core–shell electrospinning is an advanced variation of electrospinning that allows the simultaneous incorporation of two or more materials within a single fiber, thereby integrating complementary properties into a unified structure. In this technique, the core typically provides mechanical stability or controlled drug storage, while the shell offers biological functionality or tailored surface chemistry. Such multi-material fibers enable the fabrication of scaffolds that address both the structural and biological requirements of tissue engineering. Figure 2(b) shows the mechanism of core-shell electrospinning.

Incorporating core–shell electrospinning designs into ultrafine fibrous scaffolds offers a modular strategy to simultaneously address mechanical stiffness, bioactivity, and cell infiltration. A standout study fabricated a 3D multilayered patterned scaffold using coaxial electrospinning with GelMA (shell) and PDLLA (core). The structure, formed via CO_2_-assisted expansion, promoted cell infiltration, adhesion, proliferation, migration, and vascularization, accelerating wound healing in diabetic models.^115^ Similarly, PCL–collagen core–shell scaffolds supported endothelial adhesion and smooth muscle infiltration in vascular engineering contexts.^116^ An in vivo bone defect study developed scaffolds with a collagen core loaded with icariin (via chitosan microspheres), and a shell composed of collagen, PCL, and hydroxyapatite. Post-implantation results showed excellent cell attachment and bone formation, notably outperforming controls.^117^ Another study explored core–shell fibers with a PCL–PLA core and a hydroxyapatite-enriched shell, produced either as 2D sheets or 3D tubes. These scaffolds maintained mechanical integrity and supported bioactivity, including BMP-2 release and favorable cell behaviors over degradation time.^118^ A recent review emphasizes electrospun core–shell nanofibrous scaffolds as potent vehicles for delivering therapeutic agents and enhancing bone regeneration.^119^ It covers key design factors, material selection, porosity, mechanics, biodegradability, and addresses manufacturing and scalability challenges. Older yet foundational reviews underscore how core–shell architectures, generated via coaxial or emulsion electrospinning, facilitate controlled delivery of bioactives and improve cellular interactions within scaffolds.^120,121^ Table 2 summarizes the key application contexts in core-shell design and highlights the advantages.

**Table 2.** Key findings on core-shell electrospinning and their benefits.

Application context	Core-shell design	Highlighted benefit	Reference
Wound healing	GelMA shell/PDLLA core (3D patterned)	Enhanced cell infiltration & angiogenesis	^115^
Vascular tissue engineering	Collagen shell/PCL core	Supports endothelial adhesion & smooth muscle infiltration	^116^
Bone regeneration	Drug-loaded collagen-Chitosan core; PCL/collagen/HA shell	Promotes cell attachment and new bone formation	^117^
Scaffold design insights	Core–shell architecture reviews	Controlled release, customizable porosity, enhanced bioactivity	^119–121^
Tissue-specific engineering	PCL-PLA core; HA shell	Maintains mechanical strength; supports osteogenic cues	^118^

### 3D electrospinning

**Figure 4.**
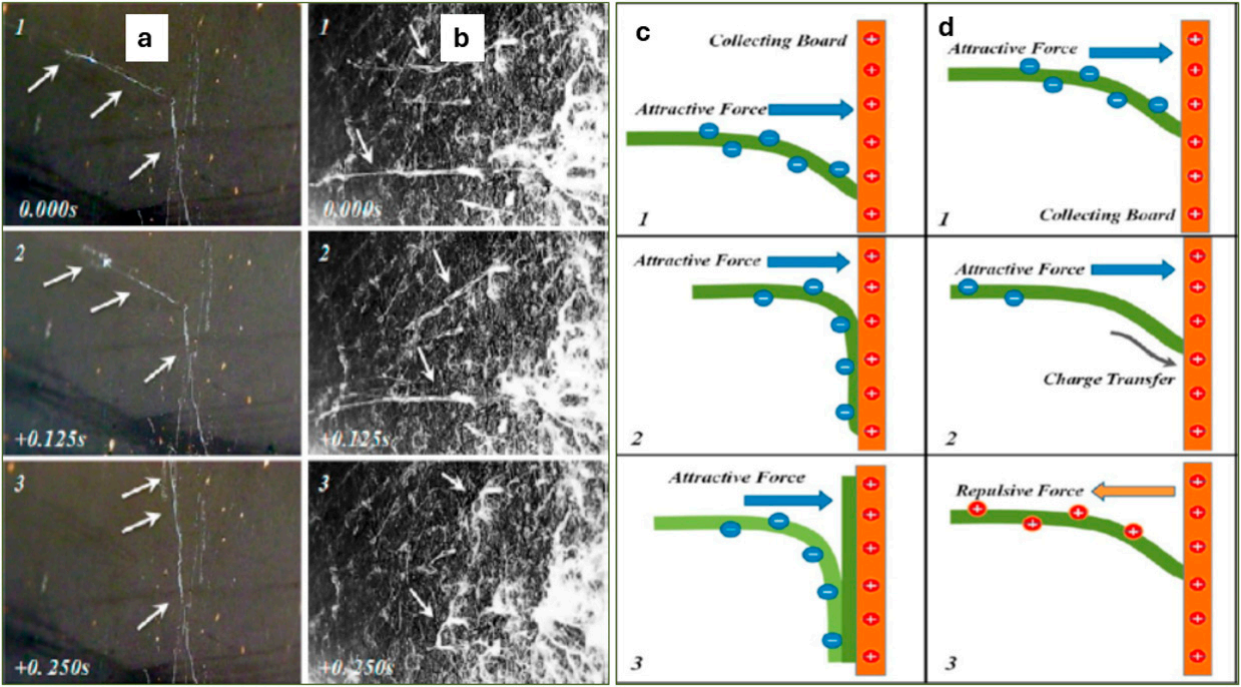
(a and b) represent continuous photography of PEG fibers in 2D and 3D electrospinning, respectively, with a 0.125 s time interval between two sequential photographs, where the white arrow represents the PEG fiber depositing on the collector. c & d are schematic diagrams of the deposition of fibers in 2D and 3D electrospinning processes. Dark green color shows the fiber depositing on the collecting board (orange color), light green indicates new fiber depositing on the old one. The black arrow represents quick charge dissipation to the collecting board, resulting in repulsive force generation between the collecting board and the depositing fiber (orange arrow)^122^.

Though wet electrospinning can physically enhance distances between the electrospun fibers and facilitate deeper penetration of cells into the interior of scaffolds to a certain degree, it cannot alter the planar orientations of the electrospun fibers. 3D zein scaffold developed by Cai et al. using novel 3D electrospinning [electrospinning by repulsive force] showed random orientation of fibers (Figure 4(a) and 4(b)) with fibers in the thickness direction as well, and the pore size is larger than 100 micrometers.^122^ They incorporated SDS (sodium dodecyl sulfate) with PEG to reduce the surface resistivity of the fibers, as low surface resistivity can cause faster charge dissipation from the fiber to the collector, resulting in an increase in repulsive force, which thus aligns the fiber in the *Z* direction (Figure 4(d)). The mechanism of this reduction in resistivity claimed by Cai et al. is that when water evaporates sulfate group of SDS concentrates on the surface of fibers, pointing towards the outside; consequently, a surface water layer is formed on Poly Ethylene Glycol (PEG) where dissociable sodium ions from SDS effectively reduce surface resistivity. They claimed that specific pore volume can be exponentially increased with decreasing surface resistivity of the fiber.

Beyond the SDS/PEG-enabled repulsive-force method described by Cai et al., other emerging fabrication strategies offer alternative pathways to three-dimensional scaffold architectures. For instance, co-electrospinning PCL with sacrificial PEO fibers creates internal voids by leaching the PEO component, significantly enhancing porosity and interconnectivity.^123^ Similarly, template-assisted electrospinning that incorporates leachable porogens (e.g., salt or sugar) also leads to enlarged pores and a more open structure upon removal of these agents.^124^ Another promising avenue involves structuring the collector itself, metallic collectors with defined holes or gaps can modify local electric fields, guiding fibers to bridge and orient in three dimensions.^125^ Collectively, these approaches, alongside the repulsive-force method, broaden the toolkit for engineering ultrafine fibrous scaffolds with enhanced structural complexity and potential for deeper cell infiltration. Table 3 summarizes and compares the key approaches through 3D electrospinning.

**Table 3.** Reported key approaches through 3D electrospinning towards tissue engineering.

Technique	Mechanism/Key modification	Benefits for 3D Scaffold & cell infiltration	References
Repulsive-force (Cai et al.)	SDS/PEG reduces surface resistivity → repulsion → *z*-direction deposition	Random 3D fiber alignment; pore sizes >100 µm	^122^
Sacrificial fiber (PCL + PEO)	Co-electrospun PEO leached post-spinning	Enhanced porosity & interconnectivity	^123^
Template-assisted (porogen leaching)	Sacrificial template within fibers, later removed	Enlarged pores, tunable architecture	^124^
Collector geometry manipulation	Structured collectors alter electric field/fiber orientation	Controlled 3D fiber orientation and bridging	^125^

In summary, 3D electrospinning techniques, including repulsive-force electrospinning, co-electrospinning with sacrificial fibers, and template- or collector-assisted methods, enable the fabrication of ultrafine fibrous scaffolds with randomly oriented fibers, larger pores, and enhanced interconnectivity, facilitating deeper cell infiltration and improved 3D scaffold architecture.

### Multimodal electrospinning

**Figure 5.**
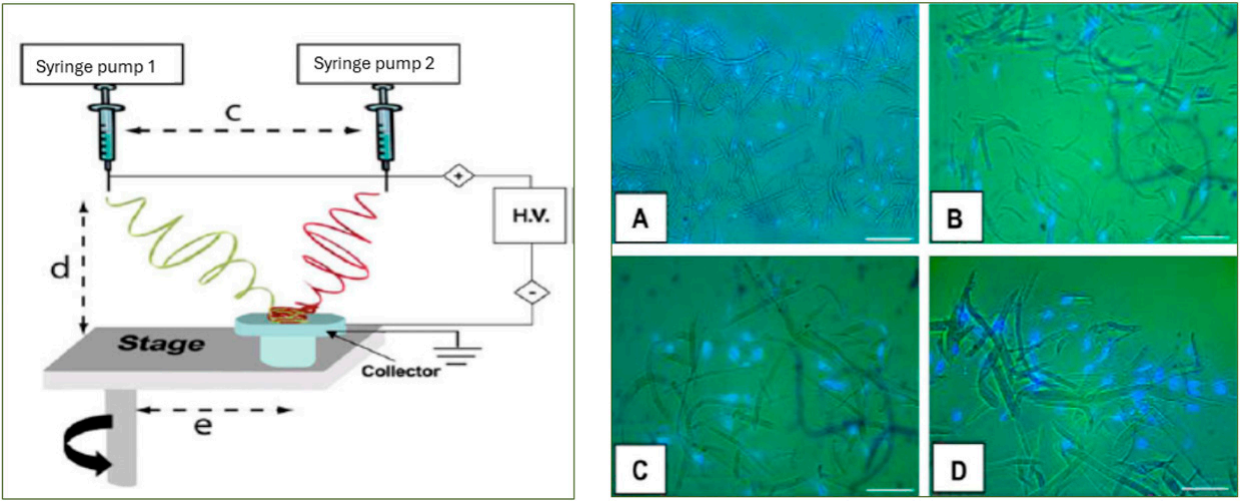
Schematic representation of multimodal electrospinning technique at left and on the right, immunofluorescence micrographs of DAPI-stained mTERT-MSC as grown after 7 days culture on Mix PCL scaffolds (fiber dia d1 = 3.3 µm and d2 = 0.6 µm). Sectioning depths from the reference surface are (a) 192 µm, (b) 128 µm, (c) 96 µm and (d) 64 µm. The scale bar is 100 µm for (a) but 50 µm for (b–d), Reprinted from,^128^ Copyright (2010), with permission from Elsevier.

The increased pore size with increasing average fiber diameter^65,126,127^ gave rise to the idea of mixing microfibers with nano-scale fibers. This is beneficial in such a way that the porosity of the structure can be simply altered by the change of nano-fiber/micro-fiber ratio; hence, it provides expected control over an important parameter. But creating a proper mixing of nano-/micro-fibers is a great challenge. If there is improper mixing, nanofibers can form sheets over microfibers and form a strong barrier for cell infiltration. This is most commonly found in multilayered electrospinning where mixing is carried out by using different scaled fiber layers. Soliman et al. proposed a modified electrospinning technique to produce bio-inspired multi-scale 3D scaffolds endowed with controlled bimodal or multimodal fiber diameter distribution with porosity of about 90%.^128^ In this method, a polymer solution of two different polymer concentrations was electrospun from two independently controlled syringe pumps on a single collector situated on a rotating mandrel (Figure 5, left). By using a rotating collector mandrel, it is possible to collect a significant number of nanofibers and microfibers alternately when the collector reaches directly under each syringe by rotation and thus maintain uniform fiber distribution. Large pore size was indicated by human bone marrow-derived mesenchymal stromal cell (mTERT-MSCs) colonization inside the bimodal 3D PCL scaffold after 7 days of culture (Figure 5, right).^128^

The concept of multimodal electrospinning, mixing fibers of different scales within a single scaffold, enables precise control over porosity and fiber architecture. Soliman et al. implemented this by using two polymer solutions from matched syringe pumps onto a rotating mandrel, achieving a controlled bimodal distribution that supported mesenchymal stromal cell colonization.^128^ Another scalable strategy purposely mingles nano- and micrometer fibers to avoid stratified layering, producing a more homogeneous scaffold structure conducive to cell infiltration.^128^

Alternatively, melt electrospinning permits the simultaneous deposition of large-diameter and nanoscale fibers without the use of solvents, facilitating full cell penetration and matrix formation while avoiding solvent-related cytotoxicity. Moreover, multi-channel electrospinning setups enable different polymer formulations to be concurrently spun, enhancing scaffold mechanical performance and improving mesenchymal stem cell adhesion through optimized fiber architecture.^129^

Overall, multimodal electrospinning combines nano- and microfibers within a single scaffold to precisely control porosity and fiber architecture, enabling enhanced cell infiltration, uniform fiber distribution, and improved scaffold mechanical performance, with techniques including dual-syringe pumps, rotating collectors, melt electrospinning, and multi-channel setups.

### Electrospinning of nanofibers onto a single microfiber

**Figure 6.**
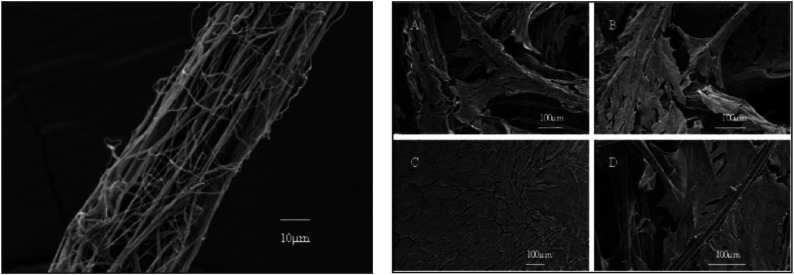
SEM image of electrospun PCL nanofibers coated single PLA microfiber (left) and Scaffolds with chondrocytes after 2 weeks of culturing (right) on PCL coated PLA microfiber scaffold with (a) 97% and (b) 95% porosities and references of (c) PCL only nanofibers and (d) PLA only microfibers, Reprinted (adapted) with permission from,^94^ Copyright (2008) American Chemical Society.

Using two spinnerets to simultaneously electrospun both nano- and microfibers is very difficult owing to the repulsive force between two fibers, and also time-consuming. Electrospinning nanofibers directly onto a single microfiber can create highly porous scaffolds with a suitable distribution of combined nano- and micro-fibers throughout the scaffold. Such a flexible technique for creating structures of variable shape, size, porosity and morphology was first introduced by Anna et al., who developed scaffold where a single PLA microfiber was coated with electrospun PCL nanofibers (Figure 6, left).^94^

**Figure 7.**
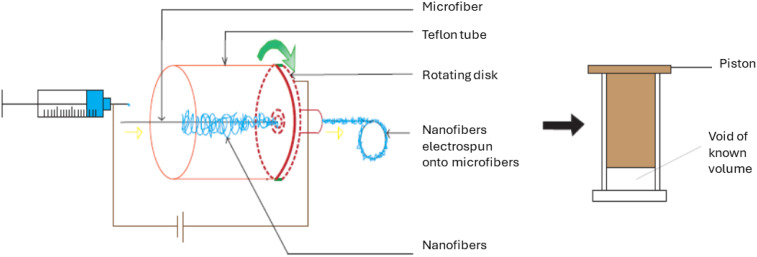
Electrospinning setup used to direct the nanofibers towards the microfiber (a) and Scaffolding device (b), redrawn from^94^.

They claimed that Scaffolds of randomly distributed mixed fibers and pore sizes of 100 µm or larger can be produced by adjusting the volume% of fibers. In their fabrication method, Poly (lactic acid)^106^ microfiber was positioned in the spinning direction from the needle via a centrally located hole in a rotating disk, on which the collector was placed. A Teflon tube was positioned between the syringe needle and the collector to confirm the collection of nanofibers on the collector and microfiber. The nanofiber-coated microfiber was wound onto a rotating wheel stationed behind the collector and then transformed into a scaffold in a separate chamber (Figure 7). Better chondrocyte cell infiltration and uniform cellular distribution were observed throughout the mixed fibers scaffold after 2 days of culture as compared to controls (Figure 6, right). Table 4 provides a summary of some key reports on electrospinning nanofibers onto single microfibers.

**Table 4.** Some major approaches to electrospinning nanofibers onto microfibers.

Approach	Polymer/Material system	Key features	Reported outcomes	Reference
Fiber diameter tuning for cell delivery	Electrospun PCL (3.4–12.1 µm fibers)	Varying fiber diameters to control pore size	Larger fibers (12.1 µm) enabled homogeneous cell penetration, improved tissue formation, and matrix deposition	^126^
Electrospinning nanofibers onto twisted microfibers	Gelatin nanofibers on silk fibroin microfibers	Hierarchical scaffold mimicking tendon/ligament fiber bundles	Improved adipose-derived mesenchymal stem cell (AMSC) attachment and distribution	^130^
Core microfiber with nanofiber wrapping	PLLA/PCL microfiber with nanofiber sheath	Coaxial integration of microfiber support and nanofibrous ECM-like surface	Balanced tensile properties with bioactive nanostructured surface	^131^
Nano/micro hybrid scaffold	P3HB or PCL nanofibers on silk fibroin (SF) yarns	Nano/micro hybrid scaffold with tightly wrapped nanofibers around silk filaments	Enhanced mechanical properties, good cytocompatibility with fibroblasts	^132^

To conclude, electrospinning nanofibers directly onto single microfibers creates highly porous scaffolds with well-distributed nano- and microfibers, allowing adjustable fiber ratios, improved cell infiltration, and uniform cellular distribution, as demonstrated using PLA microfibers coated with PCL nanofibers.

### Low temperature electrospinning

Scaffolds with four times higher porosity than standard electrospun scaffolds and with an increased pore size in the thickness direction were achieved by combining ice crystals as a removable void template with low low-temperature electrospinning process.^133^ Low temperature electrospinning, along with high relative humidity in the vicinity of the collection drum, ensures ice crystal formation, which consequently results in higher inter-fiber gaps and fibers along the *z*-direction decrease fiber mesh density. Simonet et al. in their study claimed that LTE may not be scaffolding material specific, as they found similar results for both PLGA and PEU fiber meshes.^134^ But Milleret et al. in a separate study found dissimilarities between Polyester Urethane Degrapol (DP) and Poly lactiide-co-glycolic acid (PLGA) scaffolds produced by LTE. They observed a delayed fluorescent bead adsorption in the case of DP scaffolds, which points to more fluffiness in the DP scaffold than in PLGA.^135^

More recently, Vieira et al. demonstrated a related approach by embedding glucose crystals within PEO fibers, co-electrospun alongside PLA fibers, to serve as sacrificial porogens. After PEO removal, the resulting scaffold exhibited well-defined, open pore structures (50–150 µm), which notably enabled fibroblast infiltration and colonization throughout the scaffold thickness and maintained structural integrity without triggering inflammatory responses.^136^ This pore-engineering strategy complements LTE-based methods and illustrates how combining cryogenic templating with porogen leaching can yield physiologically relevant architectures for enhanced cell infiltration.

Therefore, LTE often combined with ice crystal or porogen templating, produces scaffolds with significantly higher porosity, enlarged pores along the thickness, and well-distributed fibers in the *z*-direction, enabling enhanced cell infiltration and uniform colonization while maintaining structural integrity.

### Rapid prototyping combined with electrospinning

**Figure 8.**
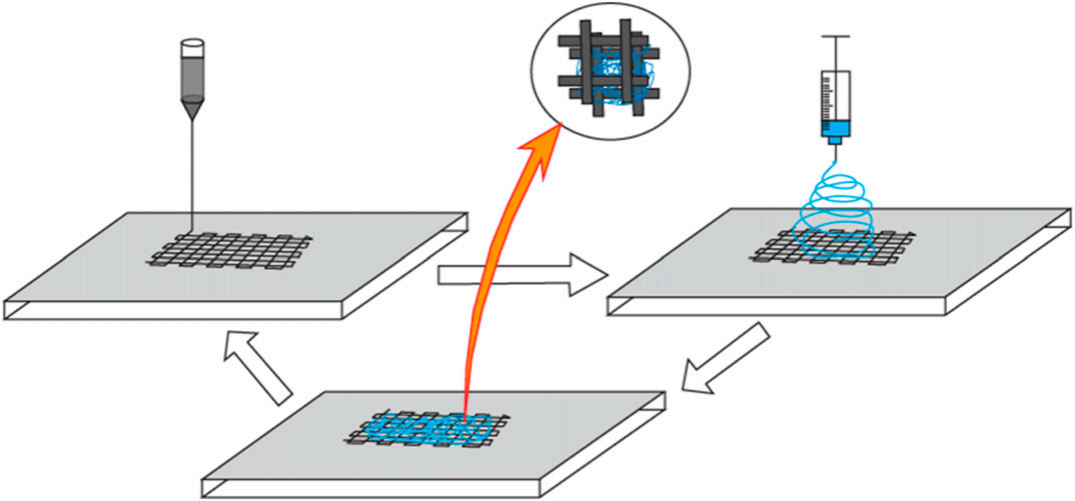
Schematic of rapid prototyping combined with electrospinning technique.

Above-discussed scaffolding techniques lack in mechanical stability and accurate designs when the need for designing complex structures arises, such as scaffolds for heart valves.^137^ In this regard, ‘Rapid prototyping’ (RP) can produce computer-designed, highly porous scaffolds of complex architecture with controlled pore size.^59^ Among several RP processes, mimicking natural body structure similar to computer tomography can be done by 3D fiber dispensing, where a computer-controlled plotter plots strands of molten polymer layer by layer from an extruder through a movable dispensing needle tip. Although it was thought that RP scaffolds would experience high cell infiltration due to having large, interconnected pores, they failed in terms of initial cell attachment due to a smooth surface and excessively large pore size compared to most cells. Moroni et al. developed Poly(ethylene oxide-terephthalate)/poly(butylene-terephtalate) (PEOT/PBT) scaffold through combined RP and electrospinning technique in a layer by layer assembly manner where macro-scaled (>100 µm) fibers deposited by RP and micro-scaled fibers deposited by electrospinning in alternating manner (Figure 8).^138^ A similar study was carried out by Kim et al., who developed a scaffold via this combined approach using Biodegradable PCL.^139^ Both studies were able to produce a fibrous 3D scaffold with enhanced cell adhesion and proliferation than single RP scaffolds without compromising mechanical stability.

More recent studies expanded on this concept, as demonstrated by the integration of FDM and electrospinning in osteochondral scaffolds.^140^ Another study engineered bilayer osteochondral scaffolds combining collagen layers with electrospun PLLA nanofibers, significantly improving cartilage formation and osteochondral regeneration.^141^ Further, a 2023 study advanced this by producing nano-hybrid RP/electrospun PCL/nHA/MWCNT scaffolds, which enhanced bone marrow stem cell proliferation and osteogenic differentiation in vitro.^142^ Together, these examples show that the RP–electrospinning hybrid approach effectively integrates mechanical robustness with tailored microenvironments for cell adhesion, infiltration, and tissue-specific regeneration. Table 5 summarizes the key combinations and outcomes reported in this regard.

**Table 5.** A summary of key material combinations and outcomes based on the combination of RP-electrospinning.

Material system	Fabrication approach	Key features	Biological outcome	References
PEOT/PBT	RP (macrofibers) + electrospinning (microfibers)	Layered scaffold with integrated macro/micro fibers, ECM-scale cues, >100 µm pores	Enhanced cell entrapment, higher ECM deposition (GAG), improved chondrocyte morphology	^138^
PCL	RP + electrospinning	Fibrous 3D scaffold, improved roughness	Better adhesion & proliferation vs. RP-only scaffolds	^139^
Collagen + PLLA	Bilayer scaffold: Collagen + electrospun PLLA nanofibers	Microporous bilayer with nanofibrous surface	Enhanced osteogenic differentiation and improved cartilage formation	^141^
PCL + nHA + MWCNTs	3D printing (RP) + electrospinning	Nano-hybrid scaffold, osteoconductive fillers	Enhanced BMSC proliferation & osteogenic differentiation	^142^

Overall, hybrid scaffolds that combine RP with electrospinning successfully bridge the gap between mechanical stability and biological performance. By leveraging macrostructural control from RP and bioactive micro/nanoscale features from electrospinning, these systems provide promising platforms for tissue engineering applications requiring both strength and functionality.

### Porogen method

**Figure 9.**
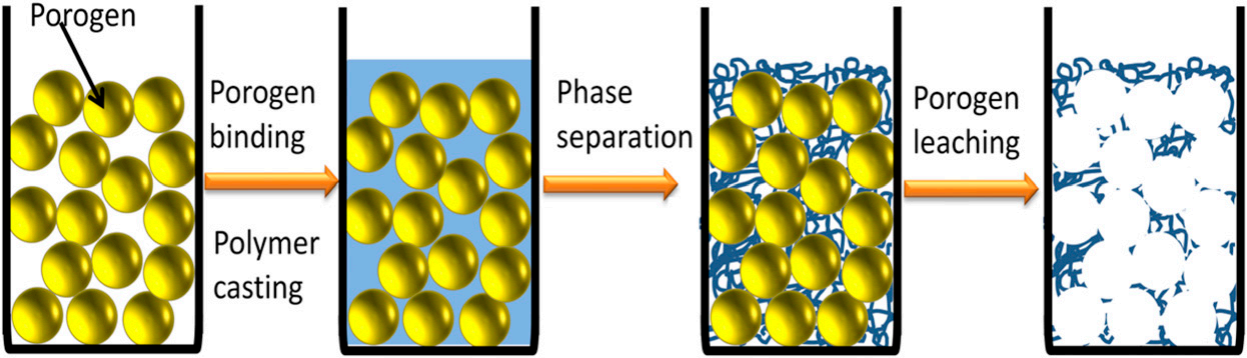
Schematic representation of particulate leaching combined with TIPS.

This method involves the incorporation of particulates such as salt or sugar spheres as a void template into either nanofiber meshes (when combined with electrospinning) or into the polymer solution (solvent casting), and later the particulates are leached out, resulting in a highly porous scaffold with interconnected pores. It is possible to create large pores with controlled pore size depending on the size of the particulate used.^67^ Lee et al. proposed a technique combining electrospinning and particulate leaching to fabricate a scaffold with both nano-scale and micro-scale structures.^66^ But to increase pore interconnectivity, produced scaffolds must be sintered which in turn may adversely affect polymer fibers. Porogen method in association with phase separation can produce interconnected macropores within the scaffolds without this adverse effect, as particulate spheres can be interconnected by applying a certain amount of heat for a certain time before incorporation of polymer/solvent mix. Such approaches developed previously suffered from poor cell adhesion due to the solid pore wall. In order to create fibrous pore morphology, Porogen leaching can be associated with TIPS (Thermally Induced Phase Separation), where particulate spheres are added onto a mold and a polymer/solvent mixture is cast into the mold and quenched.^143^ After phase separation, solvent exchange and particulate leaching are carried out by subjecting the composite to water or other chemicals that are immiscible with the polymer but soluble in solvents and lyophilized. Cheng et al. developed a 3D nanofibrous and microporous scaffold by TIPS (thermally induced phase separation) with salt leaching technique (Figure 9).^67^ They found that the micro-porous structure aided cell infiltration and multi-cellular organizations in the pores, and the nano-fibrous structures upheld cell differentiation as well as a more in-vivo-like cell-matrix adhesion.

Liumin He et al., who developed PCL-b-PLLA scaffold with a pore size of 144 ± 36 μm through salt leaching and liquid-liquid TIPS, observed abundant filopodia extension, with higher proliferation observed on nano-fibrous walled scaffold.^144^ Ma et al. created a 3D macro-porous scaffold with nano-fibrous pore walls by using a technique combining TIPS and paraffin sphere leaching.^145^ The pore interconnectivity can also be adjusted by changing heat treatment times of paraffin spheres, as longer heat-treatment times form larger bonding areas between the paraffin spheres, causing larger openings between the macropores of the scaffold.^54^ Nevertheless, there are also a few drawbacks of using paraffin spheres, as many organic solvents, e.g., tetrahydrofuran (THF), dioxane, dichloromethane, and pyridine used for polymer dissolution are soluble in paraffin to varying extents. In addition, the presence of a small amount of paraffin in the scaffolds could be unsuitable for the subsequent cell activity because of its hydrophobicity. Hence, paraffin spheres are not appropriate for porogen techniques. Macroporous and nanofibrous PLLA scaffold developed by G. Wei and P. X. Ma using Sugar sphere leaching and TIPS maintained the benefits of interconnected spherical pores, though avoiding the use of paraffin.^146^

To enhance porosity and interconnectivity in electrospun scaffolds without compromising fiber integrity, Kim et al. combined electrospinning with concurrent salt leaching, generating macroporous nanofibrous matrices that supported improved cellular infiltration, all without requiring sintering.^147^ Extending this concept, L. He et al. utilized liquid–liquid TIPS combined with salt leaching on PCL-b-PLLA. The resulting scaffolds featured ∼144 ± 36 µm interconnected pores lined with nanofibrous walls and supported robust chondrocyte culture.^144^ More recently, a hierarchical PLLA/PCL scaffold was fabricated using a single-step TIPS process followed by electrochemical deposition of hydroxyapatite, enhancing osteogenic capability and accelerating bone repair in vivo.^148^ Additionally, improved porogen integration strategies have shown a significant increase in porosity (up to ∼86.5%), enabling cellular infiltration up to 4 mm, demonstrating the critical role of controlled particle-based templating in fabricating highly penetrable scaffolds.^149^ Table 6 provides a summary of the key techniques used in this pathway.

**Table 6.** A summary of key reports on the Porogen technique in tissue engineering.

Technique	Key mechanism	Outcomes	References
Salt–Electrospinning + leaching	Salt particles embedded during electrospinning, then leached	High porosity; improved cell infiltration without fiber sintering	^147^
TIPS + salt leaching (PCL-b-PLLA)	Liquid–liquid phase separation plus salt porogen	∼144 µm interconnected pores with nanofiber walls; supports chondrocytes	^144^
TIPS + electrochemical mineralization	TIPS for structure; surface coated with hydroxyapatite	Hierarchical porosity; enhanced osteogenesis and bone repair	^148^
Salt leaching optimization	Precise incorporation and removal of salt; e.g., in electrospun	Highly porous nanofibers, improved cell growth and infiltration	^149^

In summary, particulate leaching, often combined with thermally induced phase separation (TIPS) or electrospinning, produces scaffolds with highly interconnected macropores lined with nanofibrous walls, allowing controlled pore size and interconnectivity, which enhances cell infiltration, multi-cellular organization, and in vivo-like cell–matrix interactions while maintaining fiber integrity.

### Ultra-low concentration phase separation

Jiang et al. developed an ultra-low concentration phase separation (ULCPS) method to create gelatin ultrafine fibrous scaffolds, where the loose structures of phase-separated (PS) scaffolds with larger pores and weaker fiber entanglement enabled cell infiltration and migration.^150^ They found that a lower concentration of gelatin (0.01%) can produce ultrafine fibers, and citric acid crosslinking with a certain amount of catalyst can give stability to the fibers. As compared to typical electrospun gelatin scaffolds, scaffolds produced via ULCPS showed higher cell infiltration. But these PS scaffolds undergo a large amount of shrinkage while soaked in PBS or other medium.

To mitigate the dimensional instability, subsequent studies adapted phase separation methods. For instance, a study used TIPS on gelatin/PLLA blends, yielding scaffolds with interconnected pores and enhanced resistance to shrinkage.^151^ Similarly, Perez-Puyana et al. (2020) incorporated chitosan into phase-separated gelatin scaffolds, which improved structural integrity and reduced collapse during aqueous incubation.^152^ More recently, strategies such as collagen–gelatin porous scaffold containing zinc-doped bioactive glass-ceramic nanoparticles were developed as a dermal substitute. The scaffold enhanced cell attachment, proliferation, angiogenesis, and significantly accelerated wound closure, demonstrating its potential for effective cutaneous tissue regeneration.

These advancements underscore that ULCPS is a powerful strategy for creating highly porous fibrous scaffolds, but require additional stabilization, via blending or crosslinking, to ensure practical utility in tissue engineering applications. Table 7 summarizes the key techniques and findings in this segment.

**Table 7.** A summary of the key techniques and findings relevant to ULCPS.

Material system	Method	Key features	Outcome/Limitation	References
Pure gelatin	ULCPS (0.01% gelatin) + citric acid crosslinking	Loose, ultrafine fibrous network	Excellent infiltration; significant shrinkage	^150^
Gelatin + PLLA	TIPS	Interconnected porous architecture	Improved dimensional stability	^151^
Gelatin + chitosan	Phase separation + blending	Enhanced structural robustness in aqueous environments	Stable network with maintained porosity	^152^
Collagen + Gelatin + Zn-BGC	Porous scaffold fabrication	Biocompatible; supports fibroblast/keratinocyte infiltration; promotes angiogenesis	Accelerated wound healing in vivo; optimal regeneration when loaded with fibroblasts	^153^

Overall, ULCPS enables the fabrication of ultrafine gelatin scaffolds with enhanced porosity and cell infiltration, closely mimicking native ECM architecture. To ensure their structural integrity and dimensional stability in physiological conditions, however, ULCPS scaffolds must be reinforced through robust crosslinking or blended with complementary polymers.

### Polymer–nanoparticle assisted electrospinning for ultra-thin fibers

While ULCPS has enabled the formation of ultrafine fibrous scaffolds, recent research also highlights the potential of polymer–nanoparticle hybrid systems in conventional electrospinning to achieve ultra-thin fibers with tailored properties. Incorporating nanoparticles such as silica, hydroxyapatite, or metallic nanoparticles into polymeric matrices can influence solution conductivity, charge density, and jet stability, all of which are critical parameters for fiber diameter reduction.

For example, Xia et al. (2018) prepared polyvinyl alcohol/silica (PVA/SiO_2_) hybrid fibers using a sol–gel assisted electrospinning technique for bone tissue engineering applications. At 8% PVA concentration, the fibers exhibited uniform and continuous morphology (∼543 nm diameter) with silica well distributed in the polymer matrix. Upon immersion in simulated body fluid (SBF), the fibers showed strong apatite crystal precipitation, indicating excellent in vitro bioactivity.^154^ In another study, Guo et al. designed PLLA scaffolds reinforced with halloysite nanotubes (HNT) modified with in-situ grown SiO_2_ nano-protrusions (HNT@SiO_2_). By hydrolyzing tetraethoxysilane (TEOS), irregular SiO_2_ nano-protrusions were grafted onto HNT surfaces, enhancing dispersion and interfacial adhesion with PLLA. Compared with unmodified HNT, HNT@SiO_2_ significantly improved scaffold tensile strength, modulus, hydrophilicity, and apatite-forming ability. Furthermore, the scaffolds supported stem cell responses, indicating strong potential in bone tissue engineering.^155^ In another study, a ternary nanocomposite of PEEK/PEI reinforced with TiO_2_ nanoparticles was fabricated via ultrasonication and melt-blending. PEI served as a coupling agent, ensuring homogeneous TiO_2_ dispersion through π–π stacking and hydrogen bonding. At 4 wt% TiO_2_, the scaffolds exhibited simultaneous strengthening, stiffening, and toughening, while retaining tensile properties even after steam sterilization or immersion in SBF. The composites demonstrated enhanced thermal stability, dielectric properties, tribological performance, reduced water absorption, and antibacterial activity against both Gram-positive and Gram-negative bacteria. These features highlight their promise for long-term load-bearing implant applications.^156^

To conclude, polymer–nanoparticle assisted electrospinning enables the fabrication of ultrathin, bioactive fibrous scaffolds with enhanced mechanical, thermal, and functional properties, where incorporated nanoparticles improve fiber morphology, interfacial adhesion, and cell-material interactions, making these scaffolds particularly suitable for bone tissue engineering and long-term load-bearing implant applications.

## Present scenario of cell filtration with scaffold

Cell filtration with scaffolds has come a long way in tissue engineering and regenerative medicine, which has been a significant advancement in the field of biomedical. Recent innovations are mostly focused on designing scaffolds with enhanced porosity and biocompatibility to improve cell filtration efficiency, enabling better nutrient and oxygen diffusion for cells. Lan et al.^157^ developed the gelatin-based scaffolds with the main substrate and polyamide layers so that they can make the water channels. That scaffold had the ability to affect the interfacial polymerization so that it could obtain a precise separation layer. Moreover, the membrane demonstrated better high permeability and selectivity.

Techniques like electrospinning, 3D printing, and decellularization are employed to create highly porous and customized scaffolds. Biodegradable and bioactive materials, including collagen, chitosan, and alginate, are widely used due to their ability to promote tissue growth and integration. Erben et al.^158^ used 3D printing to develop two-photon stereolithography to print up to mm-sized highly accurate 3D cell scaffolds at a very micrometer resolution. They modified the manufacturing process with two-pass printing and post-print crosslinking, maintaining a variation in Young’s moduli (from 7 to 300 kPa) are printed and quantified through AFM. The effects of different scaffold topographies on the behavior of colonizing cells were studied using mouse myoblast cells and a 3D lung microtissue replica, which was colonized with primary human lung fibroblasts. This method enables a systematic investigation of both single-cell and tissue dynamics in response to specific mechanical and biomolecular signals, with the potential to scale up for application in full organ models. Huang et al.^159^ used the biomaterials to improve the functionalities and effect of the therapeutic materials for escalating the process of immunotherapy. They also demonstrated efficient control over surface functionalization, which can impact immune cell modulation. They worked on the development of biocompatible immune cell-engaging particles (ICEp) with synthetic short DNA for fabricating the scaffolds that are capable of controlled and tunable protein loading. They intratumorally injected to ensure the safety of the chimeric antigen receptor (CAR) during T-cell therapies. Moreover, they demonstrated smart signals using an AND-gate on the CAR-T cells. This scaffold unfolded the opportunity for a versatile and modular biomaterial functionalization platform for controlled immunotherapies. Swanson et al.^160^ worked on combating craniosynostosis, a severe birth defect related to premature fusion of cranial bones due to early stem cell depletion in the suture tissue between growing bones. They developed biomaterial-based scaffolds that could maintain the stemness of cranial suture cells with an aim to reduce morbidity in craniosynostosis patients. Their findings indicate that the physical properties of synthetic scaffolds influence cell and tissue outcomes. In that study, macro-porous scaffolds with controlled spherical pores were created using a sugar porogen template method. They set up the cell-scaffold constructs in mice for up to 8 weeks and analyzed them for mineralization, vascularization, ECM composition, and gene expression. According to their findings, they showed that pore size affects cell fate: larger pores support bone formation, while smaller pores (<125 μm) maintain stemness and prevent differentiation. Similar outcomes were observed in vitro, linking scaffold pore geometry to differential cell and tissue fate. This suggests that scaffold pore size is a crucial factor in regulating mesenchymal cell fate, offering a new approach to control tissue regeneration and develop stem cell niches both in vivo and in vitro. Research from diverse applications is running current days. The scaffolds possess a variety of functionalities to keep them rolling in different applications.

Overall, cell filtration using scaffolds has advanced significantly in tissue engineering and regenerative medicine, with current research focusing on highly porous, biocompatible scaffolds that improve nutrient and oxygen diffusion, regulate cell fate, and enable precise control over cell behavior; techniques like electrospinning, 3D printing, and decellularization, combined with bioactive materials and innovative designs such as macro-porous structures, functionalized surfaces, and modular biomaterial platforms, are being used to optimize cell infiltration, tissue formation, and therapeutic applications.

## Comparative study of performance of different scaffold fabrication method

Different fabrication methods have different pros and cons for different product quality or even for the setup of the fabrication process (Table 8). A comparative study can evaluate the performance of various scaffold fabrication methods, focusing on other advantages or disadvantages of the processes. Techniques such as electrospinning, 3D printing, and solvent casting have already been discussed in the previous portions of the manuscript, and with 3D printing specifically, it can offer superior control over architecture and cell infiltration.

**Table 8.** Comparative study on different fabrication methods of the scaffolds.

Fabrication method and citation	Advantages	Disadvantages	References
Wet electrospinning (layered hydrospinning)	i. High porosity: Wet electrospinning can yield nanofibrous scaffolds with up to 99% porosity ii. Rapid production: The process is fast, allowing for the creation of more than 1 cm thick scaffolds in a relatively short period of time iii. Uniform pore size: Unlike some other techniques, wet electrospinning enables the fabrication of nanofibrous membranes with uniform pore sizes iv. Thicker porous Scaffold: Hydrospinning offers the advantage of producing thicker porous scaffolds. These scaffolds can be more substantial and provide better structural support for tissue engineering applications	i. Large pore size: The difference in pore size between hydrospun and electrospun nano-fiber scaffolds may be due to water exerting tension on fibers during hydrospinning, causing them to widen. Consequently, wet electrospun scaffolds may exhibit larger pores ii. Layer separation: Wet electrospinning can lead to layer separation within the scaffold due to tension exerted by water on the nanofibers during evacuation. Layers may separate from each other, affecting scaffold integrity	^109,161^
Wet electrospinning (layer-by-layer (L-b-L) assembly)	i. No vacuum treatment required ii. Layer-by-layer (L-b-L) assembly: Allows precise control over scaffold architecture and facilitates the incorporation of different cell types iii. Grounded liquid as collection surface; instead of using a solid collector iv. Direct cell seeding during spinning v. Controlled thickness	i. Limitations in achieving precise control over pore size ii. Complexity of multilayer assembly: Multiple layers can be intricate and time-consuming iii. Potential challenges in uniform cell distribution while seeding directly	^110,138^
Core-shell electrospinning	i. Controlled delivery of bioactive agents ii. Structural versatility iii. Enhanced cell-material interactions	i. Complex fabrication ii. Limited material combinations iii. Scale-up challenges	^119–121^
3D electrospinning	i. Allows for deeper penetration of cells into the interior of scaffolds ii. Random fiber orientation iii. The pore size in this 3D zein scaffold is larger than 100 micrometers	i. It cannot completely alter the planar orientations of electrospun fibers ii. Achieving the desired reduction in resistivity while maintaining scaffold integrity can be challenging iii. Surface resistivity reduction mechanism; further research is needed to validate this mechanism	^122,162^
Multimodal electrospinning	i. Varied porosity control by mixing microfibers with nano-scale fibers ii. The ability to adjust the ratio of micro and nanofiber provides desired control over an essential parameter in tissue engineering iii. Maintaining a uniform distribution Multimodal fiber diameter distribution and a high porosity of about 90%	i. Achieving a proper and uniform mixing of nano-/micro-fibers is a significant challenge ii. Multilayered electrospinning complexity: Different scaled fiber layers are used for mixing, achieving consistent and effective blending can be complex iii. Improperly mixed nanofibers acting as barriers can limit the penetration of cells into the scaffold	^65,126,127,163^
Electrospinning of nanofibers onto a single microfiber	i. Creates highly porous scaffolds ii. Allows for creating structures with variable shape, size, porosity, and morphology iii. Exhibited better chondrocyte cell infiltration and more uniform cellular distribution	i. Achieving proper mixing and distribution can be a challenge ii. Simultaneously electrospinning both nano- and microfibers using two spinnerets is difficult due to repulsive forces between the fibers iii. Time-consuming, especially when dealing with complex structures or large-scale production iv. Control over fiber volume Percentage can be a challenge	^94,106^
Low temperature electrospinning	i. LTE exhibits four times higher porosity compared to standard electro-spun scaffolds ii. LTE, combined with ice crystal formation, leads to an increased pore size in the thickness direction iii. Higher inter-fiber gaps, allowing for better cell penetration and tissue integration iv. Material independence: LTE may not be specific to a particular material. Studies have shown similar results for both PLGA (poly (lactic-co-glycolic acid)) and PEU (polyester urethane) fiber meshes	i. Milleret et al. Observed dissimilarities between Polyester Urethane Degrapol (DP) and Polylactide-co-glycolic acid (PLGA) scaffolds produced by LTE. ii. DP scaffolds exhibited delayed fluorescent bead adsorption, suggesting a fluffier structure compared to PLGA scaffolds	^133–135^
Rapid prototyping combined with electrospinning	i. Rapid prototyping (RP) of customized structures ii. Cell-laden materials with high precision in space iii. Highly controllable microenvironment for tissue growth iv. The layer-by-layer assembly approach in RP allows for gradual construction of the scaffold. This method facilitates the incorporation of different materials and properties	i. Fail in initial cell attachment due to their smooth surface and excessive pore size compared to most cells, i.e., ensuring proper cell adhesion remains a challenge ii. While RP scaffolds enhance cell adhesion and proliferation, there is a need to balance these benefits with mechanical stability	^59,138,139^
Porogen method	i. Highly porous scaffold with interconnected pores ii. Large pores with controlled pore size can yield a scaffold with both nano-scale and micro-scale structures when using electrospinning iii. Offers flexibility in manipulating pore size and structure, allowing for the creation of highly porous scaffolds with porosity levels up to 93% and pore diameters up to 500 μm iv. Sugar sphere leaching combined with TIPS (thermally induced phase separation) while avoiding the use of paraffin	i. Sintering may adversely affect polymer fibers ii. Poor cell adhesion due to the solid pore wall iii. It tends to result in larger pore sizes iv. Achieving accurate pore interconnectivity can be challenging v. Paraffin sphere challenges due to its hydrophobicity	^54,66,67,143–146^
Ultra-low concentration phase separation	i. Enhanced cell infiltration and migration ii. ULCPS produces ultrafine fibers, which can enhance the scaffold’s mechanical properties and mimic the natural extracellular matrix (ECM) for better cell attachment and growth iii. By crosslinking the ultrafine gelatin fibers with citric acid and a catalyst, the resulting scaffolds gain stability	i. Experience a large amount of shrinkage when soaked in phosphate-buffered saline (PBS) or other mediums	^150,164^
Polymer–nanoparticle assisted electrospinning	i. Enhanced functionality, nanoparticles can impart bioactivity, antibacterial properties, or conductivity ii. Improved mechanical properties iii. Tunable degradation and surface properties	i. Dispersion challenges, nanoparticles may agglomerate, leading to uneven fiber properties or clogging ii. Cytotoxicity risk, some nanoparticles can be harmful to cells iii. Process complexity requires careful optimization	^165^

The fabrication of fibrous scaffolds for high cell infiltration is crucial for tissue engineering and regenerative medicine applications. The best method for cell filtration depends on the specific application and requirements, such as the type of cells, the desired purity, the volume of the sample, and whether the cells need to remain viable after filtration. Wet electrospinning (Layered hydrospinning) offers high porosity (up to 99%) for efficient cell filtration, rapid production for high throughput, uniform pore size for consistent filtration, and thicker scaffolds for better structural support,^109^ making it the best option when these features are needed. Low-temperature electrospinning (LTE) boasts superior porosity compared to standard methods, increased pore size for better cell penetration, and material independence for broader applicability,^133–135^ making it the best option when these features are needed. Multimodal electrospinning offers tunable porosity but can be challenging to achieve uniform distribution.^65,126,127^ Electrospinning nanofibers onto a single microfiber creates highly porous structures, but achieving proper mixing can be difficult.^94,106^ The porogen method allows for large, controlled pores but may require additional steps for cell adhesion.^54,66,67,143–146^ Ultra-low concentration phase separation (ULCPS) enhances cell infiltration but experiences shrinkage in certain mediums.^150,164^

## Perspective, summary, and challenges

For high cell infiltration, future research should be conducted on preparing scaffolds using composite materials (as the use of both synthetic and natural biomaterials has shown better cell adhesion and mechanical stability to the scaffold^51^), either via those scaffolding routes represented in this paper or by introducing new approaches. Nowadays, the use of scaffolds is not limited to skin, bones or cartilage regeneration; its use has expanded to complex tissue engineering fields where aligned fibers or pores for proper cell-matrix interaction are necessary, such as aligned ECM fibrils in tendons, vascular and neural tissues.^166^ Many researchers tried to orient scaffold components to the cell supportive direction such as aligning the electrospun nanofibers^167,168^ or creating unidirectional tubular pores by applying directional temperature gradients while phase separating.^169^ But both efforts limit cell infiltration because the earlier effort reduces inter-fiber gaps i.e., decreased pore size, resulting in poor cell ingrowths, and the latter realizes poor cell adhesion due to solid pore walls, resulting in early cell death. Hydro spinning can create uniform cell distribution while seeded during spinning, but might not ensure proper nutrient transport to the cell in the middle of the scaffold due to its layer-by-layer assembly. So, efforts should be given to modify these scaffolds to improve cell viability and distribution by ensuring rapid cell infiltration and efficient nutrient transport. Scaffold surfaces produced via particulate leaching and TIPS can be further modified to be more cell supportive, as different cell types show varying degrees of adhesion and proliferation rates on constructs with different surface topographies.^170–172^ For example, on a surface with gradient roughness, osteoblasts show a significantly increased proliferation rate with the rise of surface roughness while fibroblasts show the contrasting trend in proliferation rate relative to surface roughness, i.e., slower rate with an enhanced roughness.^172^ Designing porous scaffolds with partly nanofibrous and somewhat smooth domains through combined particulate leaching and TIPS may perform as promising 3D matrices for co-culturing diverse cells that prefer varied surface architectures.^173^ Efforts should be made to allow active control over substrate properties by generating dynamic gradient substrates for studying directed cell migration. A recent study found that programmed erasure of substrate topography using shape memory polymer causes a rise in angular dispersion with corresponding remodeling of the actin cytoskeleton.^174^ The seeding method also has a significant impact on cell distribution throughout the scaffold. For example, Thevenot et al. claimed that cells seeded with orbital shaker result in much more uniform cell distribution throughout the scaffold than any other seeding method.^175^ Despite having an ultrafine fibrous structure, all those techniques discussed in this paper either address high cell infiltration or sacrifice mechanical properties. Thus, in the future, efforts should be made to develop methods for producing ultrafine fibrous scaffolds with good mechanical properties that can ensure high cell infiltration.

However, to create a native vivo-like 3D environment, only the preparation of 3D scaffolds is not enough. Ensuring high cell infiltration is a must. For better cell penetration, designing ultrafine fibrous scaffolds with highly porous architecture and proper control over ‘pore size’ and ‘pore interconnectivity’ is obligatory. Fibrous structures, particularly nanofibers, are necessary for proper cell adhesion and migration. Scaffold porosity in particular controls cell dissemination, nutrient supply to cells, metabolite diffusion, local pH stability and cell signaling where the pore size can have an effect on how close the cells are at the early stages of tissue growth (allowing for cell-cell communication in three dimensions) and also manage the amount of space necessary for the cells to have 3D organization in the late stages of tissue regeneration. Pore interconnectivity, on the other hand, ensures proper nutrient delivery inside the scaffold and thus prevents unexpected cell death before expected cell differentiation. So, before designing a scaffold, one should address all these factors discussed here for an effective native ECM environment.

To sum up, future scaffold design for high cell infiltration should focus on composite materials combining synthetic and natural biomaterials, optimized fiber alignment, and controlled pore architecture (size and interconnectivity) to support cell adhesion, migration, and 3D organization, while also considering surface topography, dynamic gradient substrates, and effective seeding methods to balance mechanical properties with nutrient transport and cell viability, ultimately creating scaffolds that closely mimic native ECM environments.

## Conclusions

The development of ultrafine fibrous scaffolds with enhanced cell infiltration remains a crucial challenge in tissue engineering. While nanofiber-based scaffolds provide excellent cell adhesion and mimic the extracellular matrix, their dense architecture often limits deep cell penetration. Various fabrication strategies, such as electrospinning, phase separation, and particulate leaching, have been explored to optimize scaffold porosity and interconnectivity. However, many approaches either improve cell infiltration at the expense of mechanical stability or enhance mechanical properties while restricting cell migration. Future research should focus on hybrid scaffold designs incorporating both natural and synthetic biomaterials to balance mechanical strength and bioactivity. Additionally, dynamic scaffold architectures with controlled pore size gradients and shape-memory polymers could provide adaptive microenvironments for improved cell distribution. Advanced seeding techniques, including bioreactors and orbital shaking, may further promote uniform cell infiltration. Ultimately, for scaffolds to successfully replicate native tissue environments, a multifaceted approach integrating material science, bioengineering, and cell biology is essential. By addressing the interplay between fiber morphology, pore structure, and cell-material interactions, researchers can develop next-generation scaffolds that enhance tissue regeneration outcomes. Future efforts should focus on scalable, reproducible fabrication techniques to translate these innovations into clinical applications.

## References

[1] Guan J Wang F Li Z , et al. The stimulation of the cardiac differentiation of mesenchymal stem cells in tissue constructs that mimic myocardium structure and biomechanics. Biomaterials 2011; 32: 5568–5580. 21570113 10.1016/j.biomaterials.2011.04.038 PMC4141541

[2] Yin A Zhang K McClure MJ , et al. Electrospinning collagen/chitosan/poly(L-lactic acid-co-ε-caprolactone) to form a vascular graft: mechanical and biological characterization. J Biomed Mater Res A 2013; 101: 1292–1301. 23065755 10.1002/jbm.a.34434

[3] Punnakitikashem P Truong D Menon JU , et al. Electrospun biodegradable elastic polyurethane scaffolds with dipyridamole release for small diameter vascular grafts. Acta Biomater 2014; 10: 4618–4628. 25110284 10.1016/j.actbio.2014.07.031 PMC4234112

[4] Masoumi N Annabi N Assmann A , et al. Tri-layered elastomeric scaffolds for engineering heart valve leaflets. Biomaterials 2014; 35: 7774–7785. 24947233 10.1016/j.biomaterials.2014.04.039 PMC4114056

[5] Yang F Murugan R Wang S , et al. Electrospinning of nano/micro scale poly(l-lactic acid) aligned fibers and their potential in neural tissue engineering. Biomaterials 2005; 26: 2603–2610. 15585263 10.1016/j.biomaterials.2004.06.051

[6] Yoshimoto H Shin YM Terai H , et al. A biodegradable nanofiber scaffold by electrospinning and its potential for bone tissue engineering. Biomaterials 2003; 24: 2077–2082. 12628828 10.1016/s0142-9612(02)00635-x

[7] Jang J-H Castano O Kim H-W . Electrospun materials as potential platforms for bone tissue engineering. Adv Drug Deliv Rev 2009; 61: 1065–1083. 19646493 10.1016/j.addr.2009.07.008

[8] Langer R Vacanti JP . Tissue engineering. Science 1993; 260: 920–926. 8493529 10.1126/science.8493529

[9] Jin G Prabhakaran MP Ramakrishna S . Stem cell differentiation to epidermal lineages on electrospun nanofibrous substrates for skin tissue engineering. Acta Biomater 2011; 7: 3113–3122. 21550425 10.1016/j.actbio.2011.04.017

[10] Zhang Y Zhang M . Synthesis and characterization of macroporous chitosan/calcium phosphate composite scaffolds for tissue engineering. J Biomed Mater Res 2001; 55: 304–312. 11255183 10.1002/1097-4636(20010605)55:3<304::aid-jbm1018>3.0.co;2-j

[11] Zhang Y. 3D bioprinting of vasculature network for tissue engineering. 2014, PhD thesis, University of Iowa, United States.

[12] Radhakrishnan S Nagarajan S Belaid H , et al. Fabrication of 3D printed antimicrobial polycaprolactone scaffolds for tissue engineering applications. Mater Sci Eng C 2021; 118: 111525. 10.1016/j.msec.2020.111525 33255078

[13] Ikada Y . Challenges in tissue engineering. J R Soc Interface 2006; 3: 589–601. 16971328 10.1098/rsif.2006.0124 PMC1664655

[14] Khorraminejad-Shirazi M Dorvash M Estedlal A , et al. Aging: a cell source limiting factor in tissue engineering. World J Stem Cell 2019; 11: 787–802. 10.4252/wjsc.v11.i10.787 PMC6828594 31692986

[15] Auger FA Gibot L Lacroix D . The pivotal role of vascularization in tissue engineering. Annu Rev Biomed Eng 2013; 15: 177–200. 23642245 10.1146/annurev-bioeng-071812-152428

[16] Freshney RI . Culture of specific cell types. Culture of animal cells: a manual of basic technique. New York: John Wiley & Sons, Inc., 2005.

[17] Lannutti J Reneker D Ma T , et al. Electrospinning for tissue engineering scaffolds. Mater Sci Eng C 2007; 27: 504–509.

[18] Xie Z Gao M Lobo AO , et al. 3D bioprinting in tissue engineering for medical applications: the classic and the hybrid. Polymers 2020; 12: 1717. 32751797 10.3390/polym12081717 PMC7464247

[19] Fu R-H Wang Y-C Liu S-P , et al. Decellularization and recellularization technologies in tissue engineering. Cell Transplant 2014; 23: 621–630. 24816454 10.3727/096368914X678382

[20] Placone JK Mahadik B Fisher JP . Addressing present pitfalls in 3D printing for tissue engineering to enhance future potential. APL Bioeng 2020; 4: 010901. 32072121 10.1063/1.5127860 PMC7010521

[21] Xu F Dawson C Lamb M , et al. Hydrogels for tissue engineering: addressing key design needs toward clinical translation. Front Bioeng Biotechnol 2022; 10: 849831. 35600900 10.3389/fbioe.2022.849831 PMC9119391

[22] Dhandayuthapani B Yoshida Y Maekawa T , et al. Polymeric scaffolds in tissue engineering application: a review. Int J Polym Sci 2011; 2011: 1–19.

[23] Denry I Kuhn LT . Design and characterization of calcium phosphate ceramic scaffolds for bone tissue engineering. Dent Mater 2016; 32: 43–53. 26423007 10.1016/j.dental.2015.09.008 PMC4696887

[24] Hasan A Memic A Annabi N , et al. Electrospun scaffolds for tissue engineering of vascular grafts. Acta Biomater 2014; 10: 11–25. 23973391 10.1016/j.actbio.2013.08.022 PMC3867370

[25] Ellis M Jarman-Smith M Chaudhuri JB . Bioreactor systems for tissue engineering: a four-dimensional challenge. Bioreactors for tissue engineering: principles, design and operation. Dordrecht, Netherlands: Springer, 2005, pp. 1–18.

[26] Marcacci M. Bioreactor-based, clinically oriented manufacturing of engineered tissue (BIO-COMET). University of Bologna, 2011. https://hdl.handle.net/11585/153611

[27] Nakashima M Reddi AH . The application of bone morphogenetic proteins to dental tissue engineering. Nat Biotechnol 2003; 21: 1025–1032. 12949568 10.1038/nbt864

[28] Murphy WL Peters MC Kohn DH , et al. Sustained release of vascular endothelial growth factor from mineralized poly(lactide-co-glycolide) scaffolds for tissue engineering. Biomaterials 2000; 21: 2521–2527. 11071602 10.1016/s0142-9612(00)00120-4

[29] Gerlovin L Thomson A Vailas J . Understanding the three major challenges limiting innovation in tissue engineering. Cell & Gene*, *2020*. *https://www.cellandgene.com/doc/understanding-the-three-major-challenges-limiting-innovation-in-tissue-engineering-0001 (Accessed on 02 June 2025).

[30] Jaecques SVN Van Oosterwyck H Muraru L , et al. Individualised, micro CT-based finite element modelling as a tool for biomechanical analysis related to tissue engineering of bone. Biomaterials 2004; 25: 1683–1696. 14697870 10.1016/s0142-9612(03)00516-7

[31] Hussein KH Park K-M Kang K-S , et al. Biocompatibility evaluation of tissue-engineered decellularized scaffolds for biomedical application. Mater Sci Eng C 2016; 67: 766–778. 10.1016/j.msec.2016.05.068 27287176

[32] Kasravi M Ahmadi A Babajani A , et al. Immunogenicity of decellularized extracellular matrix scaffolds: a bottleneck in tissue engineering and regenerative medicine. Biomater Res 2023; 27: 10. 36759929 10.1186/s40824-023-00348-z PMC9912640

[33] Lovett M Lee K Edwards A , et al. Vascularization strategies for tissue engineering. Tissue Eng Part B 2009; 15: 353–370. 10.1089/ten.teb.2009.0085 PMC2817665 19496677

[34] Rouwkema J Rivron NC van Blitterswijk CA . Vascularization in tissue engineering. Trends Biotechnol 2008; 26: 434–441. 18585808 10.1016/j.tibtech.2008.04.009

[35] Johnson PC Mikos AG Fisher JP , et al. Strategic directions in tissue engineering. Tissue Eng 2007; 13: 2827–2837. 18052823 10.1089/ten.2007.0335

[36] Power L Acevedo L Yamashita R , et al. Deep learning enables the automation of grading histological tissue engineered cartilage images for quality control standardization. Osteoarthr Cartil 2021; 29: 433–443. 10.1016/j.joca.2020.12.018 33422705

[37] Hunsberger J Harrysson O Shirwaiker R , et al. Manufacturing road map for tissue engineering and regenerative medicine technologies. Stem Cells Transl Med 2015; 4: 130–135. 25575525 10.5966/sctm.2014-0254 PMC4303363

[38] Kanzaki M Yamato M Yang J , et al. Dynamic sealing of lung air leaks by the transplantation of tissue engineered cell sheets. Biomaterials 2007; 28: 4294–4302. 17602737 10.1016/j.biomaterials.2007.06.009

[39] von Tigerstrom B . The legal status of engineered tissue and its implications. Cytotherapy 2014; 16: S51.

[40] de Kanter A-FJ Jongsma KR Verhaar MC , et al. The ethical implications of tissue engineering for regenerative purposes: a systematic review. Tissue Eng Part B 2023; 29: 167–187. 10.1089/ten.teb.2022.0033 PMC10122262 36112697

[41] O'Brien FJ . Biomaterials & scaffolds for tissue engineering. Mater Today 2011; 14: 88–95.

[42] Chen VJ Smith LA Ma PX . Bone regeneration on computer-designed nano-fibrous scaffolds. Biomaterials 2006; 27: 3973–3979. 16564086 10.1016/j.biomaterials.2006.02.043

[43] Abbott A . Cell culture: biology’s new dimension. Nature 2003; 424: 870–872. 12931155 10.1038/424870a

[44] Langer R Tirrell DA . Designing materials for biology and medicine. Nature 2004; 428: 487–492. 15057821 10.1038/nature02388

[45] Lee J Cuddihy MJ Kotov NA . Three-dimensional cell culture matrices: state of the art. Tissue Eng Part B 2008; 14: 61–86. 10.1089/teb.2007.0150 18454635

[46] Pampaloni F Reynaud EG Stelzer EH . The third dimension bridges the gap between cell culture and live tissue. Nat Rev Mol Cell Biol 2007; 8: 839–845. 17684528 10.1038/nrm2236

[47] Griffith LG Swartz MA . Capturing complex 3D tissue physiology in vitro. Nat Rev Mol Cell Biol 2006; 7: 211–224. 16496023 10.1038/nrm1858

[48] Haycock JW . 3D cell culture: a review of current approaches and techniques. Methods Mol Biol 2011; 695: 1–15. 21042962 10.1007/978-1-60761-984-0_1

[49] Du C Cui FZ Feng QL , et al. Tissue response to nano-hydroxyapatite/collagen composite implants in marrow cavity. J Biomed Mater Res 1998; 42: 540–548. 9827677 10.1002/(sici)1097-4636(19981215)42:4<540::aid-jbm9>3.0.co;2-2

[50] Liao SS Cui FZ Zhang W , et al. Hierarchically biomimetic bone scaffold materials: nano-HA/collagen/PLA composite. J Biomed Mater Res B Appl Biomater 2004; 69: 158–165. 15116405 10.1002/jbm.b.20035

[51] Wei G Ma PX . Structure and properties of nano-hydroxyapatite/polymer composite scaffolds for bone tissue engineering. Biomaterials 2004; 25: 4749–4757. 15120521 10.1016/j.biomaterials.2003.12.005

[52] Wang H Li Y Zuo Y , et al. Biocompatibility and osteogenesis of biomimetic nano-hydroxyapatite/polyamide composite scaffolds for bone tissue engineering. Biomaterials 2007; 28: 3338–3348. 17481726 10.1016/j.biomaterials.2007.04.014

[53] Kim HW Kim HE Salih V . Stimulation of osteoblast responses to biomimetic nanocomposites of gelatin-hydroxyapatite for tissue engineering scaffolds. Biomaterials 2005; 26: 5221–5230. 15792549 10.1016/j.biomaterials.2005.01.047

[54] Liu X Smith LA Hu J , et al. Biomimetic nanofibrous gelatin/apatite composite scaffolds for bone tissue engineering. Biomaterials 2009; 30: 2252–2258. 19152974 10.1016/j.biomaterials.2008.12.068 PMC2679864

[55] Zhang K Qian Y Wang H , et al. Genipin-crosslinked silk fibroin/hydroxybutyl chitosan nanofibrous scaffolds for tissue-engineering application. J Biomed Mater Res A 2010; 95: 870–881. 20824649 10.1002/jbm.a.32895

[56] Galbraith CG Sheetz MP . Forces on adhesive contacts affect cell function. Curr Opin Cell Biol 1998; 10: 566–571. 9818165 10.1016/s0955-0674(98)80030-6

[57] Hartgerink JD Beniash E Stupp SI . Self-assembly and mineralization of peptide-amphiphile nanofibers. Science 2001; 294: 1684–1688. 11721046 10.1126/science.1063187

[58] Kuntz RM Saltzman WM . Neutrophil motility in extracellular matrix gels: mesh size and adhesion affect speed of migration. Biophys J 1997; 72: 1472–1480. 9138592 10.1016/S0006-3495(97)78793-9 PMC1184529

[59] Abrams GA Goodman SL Nealey PF , et al. Nanoscale topography of the basement membrane underlying the corneal epithelium of the rhesus macaque. Cell Tissue Res 2000; 299: 39–46. 10654068 10.1007/s004419900074

[60] Miller DC Thapa A Haberstroh KM , et al. Endothelial and vascular smooth muscle cell function on poly(lactic-co-glycolic acid) with nano-structured surface features. Biomaterials 2004; 25: 53–61. 14580908 10.1016/s0142-9612(03)00471-x

[61] Benya PD Shaffer JD . Dedifferentiated chondrocytes reexpress the differentiated collagen phenotype when cultured in agarose gels. Cell 1982; 30: 215–224. 7127471 10.1016/0092-8674(82)90027-7

[62] Puppi D Chiellini F Piras AM , et al. Polymeric materials for bone and cartilage repair. Prog Polym Sci 2010; 35: 403–440.

[63] Zhang R Ma PX . Synthetic nano-fibrillar extracellular matrices with predesigned macroporous architectures. J Biomed Mater Res 2000; 52: 430–438. 10951385 10.1002/1097-4636(200011)52:2<430::aid-jbm25>3.0.co;2-l

[64] Chen VJ Ma PX . Nano-fibrous poly(L-lactic acid) scaffolds with interconnected spherical macropores. Biomaterials 2004; 25: 2065–2073. 14741621 10.1016/j.biomaterials.2003.08.058

[65] Pham QP Sharma U Mikos AG . Electrospun poly(epsilon-caprolactone) microfiber and multilayer nanofiber/microfiber scaffolds: characterization of scaffolds and measurement of cellular infiltration. Biomacromolecules 2006; 7: 2796–2805. 17025355 10.1021/bm060680j

[66] Lee YH Lee JH An IG , et al. Electrospun dual-porosity structure and biodegradation morphology of Montmorillonite reinforced PLLA nanocomposite scaffolds. Biomaterials 2005; 26: 3165–3172. 15603811 10.1016/j.biomaterials.2004.08.018

[67] Cheng K Kisaalita WS . Exploring cellular adhesion and differentiation in a micro-/nano-hybrid polymer scaffold. Biotechnol Prog 2010; 26: 838–846. 20196160 10.1002/btpr.391

[68] Curtis A Riehle M . Tissue engineering: the biophysical background. Phys Med Biol 2001; 46: R47–R65. 11324976 10.1088/0031-9155/46/4/201

[69] Coombes AG Rizzi SC Williamson M , et al. Precipitation casting of polycaprolactone for applications in tissue engineering and drug delivery. Biomaterials 2004; 25: 315–325. 14585719 10.1016/s0142-9612(03)00535-0

[70] Bissell MJ Rizki A Mian IS . Tissue architecture: the ultimate regulator of breast epithelial function. Curr Opin Cell Biol 2003; 15: 753–762. 14644202 10.1016/j.ceb.2003.10.016 PMC2933200

[71] Debnath J Brugge JS . Modelling glandular epithelial cancers in three-dimensional cultures. Nat Rev Cancer 2005; 5: 675–688. 16148884 10.1038/nrc1695

[72] Paszek MJ Weaver VM . The tension mounts: mechanics meets morphogenesis and malignancy. J Mammary Gland Biol Neoplasia 2004; 9: 325–342. 15838603 10.1007/s10911-004-1404-x

[73] Wozniak MA Desai R Solski PA , et al. ROCK-generated contractility regulates breast epithelial cell differentiation in response to the physical properties of a three-dimensional collagen matrix. J Cell Biol 2003; 163: 583–595. 14610060 10.1083/jcb.200305010 PMC2173660

[74] Zegers MM O'Brien LE Yu W , et al. Epithelial polarity and tubulogenesis in vitro. Trends Cell Biol 2003; 13: 169–176. 12667754 10.1016/s0962-8924(03)00036-9

[75] Langer R . Biomaterials and biomedical engineering. Chem Eng Sci 1995; 50: 4109–4121.

[76] Woodfield TB Malda J de Wijn J , et al. Design of porous scaffolds for cartilage tissue engineering using a three-dimensional fiber-deposition technique. Biomaterials 2004; 25: 4149–4161. 15046905 10.1016/j.biomaterials.2003.10.056

[77] DeWitt A Iida T Lam HY , et al. Affinity regulates spatial range of EGF receptor autocrine ligand binding. Dev Biol 2002; 250: 305–316. 12376105

[78] Ribeiro A Vargo S Powell EM , et al. Substrate three-dimensionality induces elemental morphological transformation of sensory neurons on a physiologic timescale. Tissue Eng 2012; 18: 93–102. 10.1089/ten.tea.2011.0221 PMC3246411 21910606

[79] Freed LE Martin I Vunjak-Novakovic G . Frontiers in tissue engineering. In vitro modulation of chondrogenesis. Clin Orthop Relat Res 1999: S46–S58. 10546635

[80] Khang G Lee SJ Kim MS , et al. Biomaterials: tissue engineering and scaffolds. In: Encyclopedia of Medical Devices and Instrumentation. John Wiley & Sons, Inc., 2006.

[81] Chaignaud B Langer R Vacanti J . The history of tissue engineering using synthetic biodegradable polymer scaffolds and cells. In: Atala A Mooney D (eds). Synthetic Biodegradable Polymer Scaffolds. Birkhäuser Boston, 1997, pp. 1–14.

[82] Harrison K . Biomedical polymers: introduction to polymeric scaffolds for tissue Engineering. Wood Head Punlishing Limited, 2007, pp. 1–32.

[83] Woo KM Chen VJ Ma PX . Nano-fibrous scaffolding architecture selectively enhances protein adsorption contributing to cell attachment. J Biomed Mater Res A 2003; 67: 531–537. 14566795 10.1002/jbm.a.10098

[84] Thomas JW Michael CW Janice LM , et al. Nano-biotechnology: carbon nanofibres as improved neural and orthopaedic implants. Nanotechnology 2004; 15: 48–54. 10.1088/0957-4484/15/1/009 34911207

[85] Nair LS Bhattacharyya S Laurencin CT . Nanotechnology and tissue engineering: the scaffold based approach. In: Nanotechnologies for the Life Sciences. Wiley-VCH Verlag GmbH & Co. KGaA, 2007.

[86] Gupta D Venugopal J Prabhakaran MP , et al. Aligned and random nanofibrous substrate for the in vitro culture of Schwann cells for neural tissue engineering. Acta Biomater 2009; 5: 2560–2569. 19269270 10.1016/j.actbio.2009.01.039

[87] Shanmugasundaram S Griswold KA Prestigiacomo CJ , et al. Applications of electrospinning: tissue engineering scaffolds and drug delivery system. In: Bioengineering Conference, 2004 Proceedings of the IEEE 30th Annual Northeast, Springfield, MA, USA, 17-18 April 2004, pp. 140–141.

[88] Bondar B Fuchs S Motta A , et al. Functionality of endothelial cells on silk fibroin nets: comparative study of micro- and nanometric fibre size. Biomaterials 2008; 29: 561–572. 17942151 10.1016/j.biomaterials.2007.10.002

[89] Li WJ Jiang YJ Tuan RS . Chondrocyte phenotype in engineered fibrous matrix is regulated by fiber size. Tissue Eng 2006; 12: 1775–1785. 16889508 10.1089/ten.2006.12.1775

[90] Sun T Norton D McKean RJ , et al. Development of a 3D cell culture system for investigating cell interactions with electrospun fibers. Biotechnol Bioeng 2007; 97: 1318–1328. 17171721 10.1002/bit.21309

[91] Kwon IK Kidoaki S Matsuda T . Electrospun nano-to microfiber fabrics made of biodegradable copolyesters: structural characteristics, mechanical properties and cell adhesion potential. Biomaterials 2005; 26: 3929–3939. 15626440 10.1016/j.biomaterials.2004.10.007

[92] Tuzlakoglu K Bolgen N Salgado AJ , et al. Nano- and micro-fiber combined scaffolds: a new architecture for bone tissue engineering. J Mater Sci Mater Med 2005; 16: 1099–1104. 16362207 10.1007/s10856-005-4713-8

[93] Anik HR Tushar SI Mahmud S , et al. Into the revolution of NanoFusion: Merging high performance and aesthetics by nanomaterials in textile finishes. Adv Mater Interfac 2024; 12: 2400368.

[94] Thorvaldsson A Stenhamre H Gatenholm P , et al. Electrospinning of highly porous scaffolds for cartilage regeneration. Biomacromolecules 2008; 9: 1044–1049. 18260633 10.1021/bm701225a

[95] Powell HM Boyce ST . Fiber density of electrospun gelatin scaffolds regulates morphogenesis of dermal-epidermal skin substitutes. J Biomed Mater Res A 2008; 84: 1078–1086. 17685398 10.1002/jbm.a.31498

[96] Telemeco TA Ayres C Bowlin GL , et al. Regulation of cellular infiltration into tissue engineering scaffolds composed of submicron diameter fibrils produced by electrospinning. Acta Biomater 2005; 1: 377–385. 16701819 10.1016/j.actbio.2005.04.006

[97] Birgersdotter A Sandberg R Ernberg I . Gene expression perturbation in vitro—a growing case for three-dimensional (3D) culture systems. Semin Cancer Biol 2005; 15: 405–412. 16055341 10.1016/j.semcancer.2005.06.009

[98] Ma PX . Biomimetic materials for tissue engineering. Adv Drug Deliv Rev 2008; 60: 184–198. 18045729 10.1016/j.addr.2007.08.041 PMC2271038

[99] Gomes ME Holtorf HL Reis RL , et al. Influence of the porosity of starch-based fiber mesh scaffolds on the proliferation and osteogenic differentiation of bone marrow stromal cells cultured in a flow perfusion bioreactor. Tissue Eng 2006; 12: 801–809. 16674293 10.1089/ten.2006.12.801

[100] Holtorf HL Datta N Jansen JA , et al. Scaffold mesh size affects the osteoblastic differentiation of seeded marrow stromal cells cultured in a flow perfusion bioreactor. J Biomed Mater Res A 2005; 74: 171–180. 15965910 10.1002/jbm.a.30330

[101] Mygind T Stiehler M Baatrup A , et al. Mesenchymal stem cell ingrowth and differentiation on coralline hydroxyapatite scaffolds. Biomaterials 2007; 28: 1036–1047. 17081601 10.1016/j.biomaterials.2006.10.003

[102] Grad S Kupcsik L Gorna K , et al. The use of biodegradable polyurethane scaffolds for cartilage tissue engineering: potential and limitations. Biomaterials 2003; 24: 5163–5171. 14568433 10.1016/s0142-9612(03)00462-9

[103] Chang H-I Wang Y . In: Eberli D. (ed) Cell responses to surface and architecture of tissue engineering scaffolds. In: Regenerative medicine and tissue engineering-cells and biomaterials. InTechOpen, 2011. doi:10.5772/21983.

[104] Raghunath J Rollo J Sales KM , et al. Biomaterials and scaffold design: key to tissue-engineering cartilage. Biotechnol Appl Biochem 2007; 46: 73–84. 17227284 10.1042/BA20060134

[105] Quirk RA France RM Shakesheff KM , et al. Supercritical fluid technologies and tissue engineering scaffolds. Curr Opin Solid State Mater Sci 2004; 8: 313–321.

[106] Karageorgiou V Kaplan D . Porosity of 3D biomaterial scaffolds and osteogenesis. Biomaterials 2005; 26: 5474–5491. 15860204 10.1016/j.biomaterials.2005.02.002

[107] Nam YS Park TG . Porous biodegradable polymeric scaffolds prepared by thermally induced phase separation. J Biomed Mater Res 1999; 47: 8–17. 10400875 10.1002/(sici)1097-4636(199910)47:1<8::aid-jbm2>3.0.co;2-l

[108] Botchwey EA Dupree MA Pollack SR , et al. Tissue engineered bone: measurement of nutrient transport in three-dimensional matrices. J Biomed Mater Res A 2003; 67: 357–367. 14517896 10.1002/jbm.a.10111

[109] Tzezana R Zussman E Levenberg S . A layered ultra-porous scaffold for tissue engineering, created via a hydrospinning method. Tissue Eng C Methods 2008; 14: 281–288. 10.1089/ten.tec.2008.0201 18781888

[110] Xiaochuan YHW. Electrospun functional nanofibrous scaffolds for tissue engineering. In: *Tissue Engineering*, 2010. IntechOpen. doi:10.5772/8595.

[111] Wang H Fu X Ratnamani MPC . Biomimetic nanofiber-enabled rapid creation of skin grafts. In: Thomas J. Webster (ed) Nanomedicine. Elsevier, 2023, pp. 261–296. 10.1016/B978-0-12-818627-5.00009-9

[112] Mukasheva F Adilova L Dyussenbinov A , et al. Optimizing scaffold pore size for tissue engineering: insights across various tissue types. Front Bioeng Biotechnol 2024; 12: 1444986. 39600888 10.3389/fbioe.2024.1444986 PMC11588461

[113] Wang H Xu X Qin Y , et al. Wet-electrospun porous freeform scaffold enhances colonisation of cells. Mater Today Bio 2025; 33: 101997. 10.1016/j.mtbio.2025.101997 PMC12221573 40605991

[114] Rafiei M Jooybar E Abdekhodaie MJ , et al. Construction of 3D fibrous PCL scaffolds by coaxial electrospinning for protein delivery. Mater Sci Eng C 2020; 113: 110913. 10.1016/j.msec.2020.110913 32487419

[115] Li J Zhang T Pan M , et al. Nanofiber/hydrogel core–shell scaffolds with three-dimensional multilayer patterned structure for accelerating diabetic wound healing. J Nanobiotechnol 2022; 20: 28. 10.1186/s12951-021-01208-5 PMC8742387 34998407

[116] Duan N Geng X Ye L , et al. A vascular tissue engineering scaffold with core–shell structured nano-fibers formed by coaxial electrospinning and its biocompatibility evaluation. Biomed Mater 2016; 11: 035007. 27206161 10.1088/1748-6041/11/3/035007

[117] Zhao H Tang J Zhou D , et al. Electrospun icariin-loaded core-shell collagen, polycaprolactone, hydroxyapatite composite scaffolds for the repair of rabbit tibia bone defects. Int J Nanomed 2020; 15: 3039–3056. 10.2147/IJN.S238800 PMC7200251 32431500

[118] Kareem MM Hodgkinson T Sanchez MS , et al. Hybrid core–shell scaffolds for bone tissue engineering. Biomed Mater 2019; 14: 025008. 30609417 10.1088/1748-605X/aafbf1

[119] Ang BC Nam HY Abdullah MF , et al. A review on advances and challenges in core-shell scaffolds for bone tissue engineering: design, fabrication, and clinical translation. Macromol Rapid Commun 2025; 46: 2400620. 10.1002/marc.202400620 39489721

[120] Sperling LE Reis KP Pranke P , et al. Advantages and challenges offered by biofunctional core–shell fiber systems for tissue engineering and drug delivery. Drug Discov Today 2016; 21: 1243–1256. 27155458 10.1016/j.drudis.2016.04.024

[121] Zhang H Zhao C Zhao Y , et al. Electrospinning of ultrafine core/shell fibers for biomedical applications. Sci China Chem 2010; 53: 1246–1254.

[122] Cai S Xu H Jiang Q , et al. Novel 3D electrospun scaffolds with fibers oriented randomly and evenly in three dimensions to closely mimic the unique architectures of extracellular matrices in soft tissues: fabrication and mechanism study. Langmuir 2013; 29: 2311–2318. 23390966 10.1021/la304414j

[123] Gao Q Gu H Zhao P , et al. Fabrication of electrospun nanofibrous scaffolds with 3D controllable geometric shapes. Mater Des 2018; 157: 159–169.

[124] Chen Y Shafiq M Liu M , et al. Advanced fabrication for electrospun three-dimensional nanofiber aerogels and scaffolds. Bioact Mater 2020; 5: 963–979. 32671291 10.1016/j.bioactmat.2020.06.023 PMC7334396

[125] Götz A Grabow N Illner S . Fiber orientation on 3D structured collectors for electrospinning. In: Current Directions in Biomedical Engineering. De Gruyter, 2022, pp. 364–367.

[126] Balguid A Mol A van Marion MH , et al. Tailoring fiber diameter in electrospun poly(epsilon-caprolactone) scaffolds for optimal cellular infiltration in cardiovascular tissue engineering. Tissue Eng 2009; 15: 437–444. 10.1089/ten.tea.2007.0294 18694294

[127] Eichhorn SJ Sampson WW . Statistical geometry of pores and statistics of porous nanofibrous assemblies. J R Soc Interface 2005; 2: 309–318. 16849188 10.1098/rsif.2005.0039 PMC1578270

[128] Soliman S Pagliari S Rinaldi A , et al. Multiscale three-dimensional scaffolds for soft tissue engineering via multimodal electrospinning. Acta Biomater 2010; 6: 1227–1237. 19887125 10.1016/j.actbio.2009.10.051

[129] Goreninskii S Chernova U Prosetskaya E , et al. Single-channel and multi-channel electrospinning for the fabrication of PLA/PCL tissue engineering scaffolds: comparative study of the materials physicochemical and biological properties. Cornell University 2024. 10.48550/arXiv.2403.00767

[130] Hajiabbas M Alemzadeh I Vossoughi M . A porous hydrogel-electrospun composite scaffold made of oxidized alginate/gelatin/silk fibroin for tissue engineering application. Carbohydr Polym 2020; 245: 116465. 32718603 10.1016/j.carbpol.2020.116465

[131] Su Y Toftdal MS Le Friec A , et al. 3D electrospun synthetic extracellular matrix for tissue regeneration. Small Sci 2021; 1: 2100003. 40213056 10.1002/smsc.202100003 PMC11935887

[132] Naghashzargar E Farè S Catto V , et al. Nano/micro hybrid scaffold of PCL or P3HB nanofibers combined with silk fibroin for tendon and ligament tissue engineering. J Appl Biomater Funct Mater 2015; 13: 156–168. 10.5301/jabfm.5000216 25589157

[133] Solchaga LA Penick KJ Welter JF . Chondrogenic differentiation of bone marrow-derived mesenchymal stem cells: tips and tricks. Methods Mol Biol 2011; 698: 253–278. 21431525 10.1007/978-1-60761-999-4_20 PMC3106977

[134] Simonet M Schneider OD Neuenschwander P , et al. Ultraporous 3D polymer meshes by low-temperature electrospinning: use of ice crystals as a removable void template. Polym Eng Sci 2007; 47: 2020–2026.

[135] Milleret V Simonet M Bittermann AG , et al. Cyto- and hemocompatibility of a biodegradable 3D-scaffold material designed for medical applications. J Biomed Mater Res B Appl Biomater 2009; 91: 109–121. 19360887 10.1002/jbm.b.31379

[136] Vieira T Afonso AF Correia C , et al. Electrospun poly (lactic acid) membranes with defined pore size to enhance cell infiltration. Heliyon 2024; 10: e36091. 39224377 10.1016/j.heliyon.2024.e36091 PMC11367500

[137] Chen R Morsi Y Patel S , et al. A novel approach via combination of electrospinning and FDM for tri-leaflet heart valve scaffold fabrication. Front Mater Sci China 2009; 3: 359–366.

[138] Moroni L Schotel R Hamann D , et al. 3D fiber-deposited electrospun integrated scaffolds enhance cartilage tissue formation. Adv Funct Mater 2008; 18: 53–60.

[139] Kim G Son J Park S , et al. Hybrid process for fabricating 3D hierarchical scaffolds combining rapid prototyping and electrospinning. Macromol Rapid Commun 2008; 29: 1577–1581.

[140] Schumann D Ekaputra AK Lam CX , et al. Biomaterials/scaffolds. In: Hauser H Fussenegger M (eds). Tissue Engineering Methods in Molecular Medicine™. Humana Press, 2007.

[141] Fu J-N Wang X Yang M , et al. Scaffold-based tissue engineering strategies for osteochondral repair. Front Bioeng Biotechnol 2022; 9: 812383. 35087809 10.3389/fbioe.2021.812383 PMC8787149

[142] Cao Y Sun L Liu Z , et al. 3D printed-electrospun PCL/hydroxyapatite/MWCNTs scaffolds for the repair of subchondral bone. Regen Biomater 2023; 10: rbac104. 36683741 10.1093/rb/rbac104 PMC9847519

[143] Ma PX Zhang R . Synthetic nano-scale fibrous extracellular matrix. J Biomed Mater Res 1999; 46: 60–72. 10357136 10.1002/(sici)1097-4636(199907)46:1<60::aid-jbm7>3.0.co;2-h

[144] He L Liu B Xipeng G , et al. Microstructure and properties of nano-fibrous PCL-b-PLLA scaffolds for cartilage tissue engineering. Eur Cell Mater 2009; 18: 63–74. 19859871 10.22203/ecm.v018a06

[145] Liu X Ma PX . Phase separation, pore structure, and properties of nanofibrous gelatin scaffolds. Biomaterials 2009; 30: 4094–4103. 19481080 10.1016/j.biomaterials.2009.04.024 PMC2744837

[146] Wei G Ma PX . Macroporous and nanofibrous polymer scaffolds and polymer/bone-like apatite composite scaffolds generated by sugar spheres. J Biomed Mater Res A 2006; 78: 306–315. 16637043 10.1002/jbm.a.30704

[147] Kim TG Chung HJ Park TG . Macroporous and nanofibrous hyaluronic acid/collagen hybrid scaffold fabricated by concurrent electrospinning and deposition/leaching of salt particles. Acta Biomater 2008; 4: 1611–1619. 18640884 10.1016/j.actbio.2008.06.008

[148] Nie W Gao Y McCoul DJ , et al. Rapid mineralization of hierarchical poly (l-lactic acid)/poly (ε-caprolactone) nanofibrous scaffolds by electrodeposition for bone regeneration. Int J Nanomed 2019; 14: 3929–3941. 10.2147/IJN.S205194 PMC6549790 31213809

[149] Rafiq M Khan RS Wani TU , et al. Improvisations to electrospinning techniques and ultrasonication process to nanofibers for high porosity: ideal for cell infiltration and tissue integration. Mater Today Commun 2023; 35: 105695.

[150] Jiang Q Xu H Cai S , et al. Ultrafine fibrous gelatin scaffolds with deep cell infiltration mimicking 3D ECMs for soft tissue repair. J Mater Sci Mater Med 2014; 25: 1789–1800. 24728742 10.1007/s10856-014-5208-2

[151] Rarima R Unnikrishnan G . Poly (lactic acid)/gelatin foams by non-solvent induced phase separation for biomedical applications. Polym Degrad Stabil 2020; 177: 109187.

[152] Perez-Puyana V Rubio-Valle JF Jiménez-Rosado M , et al. Alternative processing methods of hybrid porous scaffolds based on gelatin and chitosan. J Mech Behav Biomed Mater 2020; 102: 103472. 31605930 10.1016/j.jmbbm.2019.103472

[153] Sharifi E Yousefiasl S Laderian N , et al. Cell-loaded genipin cross-linked collagen/gelatin skin substitute adorned with zinc-doped bioactive glass-ceramic for cutaneous wound regeneration. Int J Biol Macromol 2023; 251: 125898. 37479201 10.1016/j.ijbiomac.2023.125898

[154] Xia S-H Teng S-H Wang P . Synthesis of bioactive polyvinyl alcohol/silica hybrid fibers for bone regeneration. Mater Lett 2018; 213: 181–184.

[155] Guo W Xu L Feng P , et al. In-situ growth of silica nano-protrusions on halloysite nanotubes for interfacial reinforcement in polymer/halloysite scaffolds. Appl Surf Sci 2020; 513: 145772.

[156] Díez-Pascual AM Díez-Vicente AL . Nano-TiO2 reinforced PEEK/PEI blends as biomaterials for load-bearing implant applications. ACS Appl Mater Interfaces 2015; 7: 5561–5573. 25706225 10.1021/acsami.5b00210

[157] Lan H Li P Wang H , et al. Construction of a gelatin scaffold with water channels for preparing a high performance nanofiltration membrane. Separ Purif Technol 2021; 264: 118391.

[158] Erben A Hörning M Hartmann B , et al. Precision 3D-printed cell scaffolds mimicking native tissue composition and mechanics. Adv Healthcare Mater 2020; 9: 2000918. 10.1002/adhm.202000918 33025765

[159] Huang X Williams JZ Chang R , et al. DNA scaffolds enable efficient and tunable functionalization of biomaterials for immune cell modulation. Nat Nanotechnol 2021; 16: 214–223. 33318641 10.1038/s41565-020-00813-z PMC7878327

[160] Swanson WB Omi M Zhang Z , et al. Macropore design of tissue engineering scaffolds regulates mesenchymal stem cell differentiation fate. Biomaterials 2021; 272: 120769. 33798961 10.1016/j.biomaterials.2021.120769 PMC8068670

[161] Yang S Leong K-F Du Z , et al. The design of scaffolds for use in tissue engineering. Part I. Traditional factors. Tissue Eng 2001; 7: 679–689. 11749726 10.1089/107632701753337645

[162] Bonino C Efimenko K Jeong S , et al. Three-dimensional electrospun alginate nanofiber mats via tailored charge repulsions. Small 2012; 8: 1928–1936. 22461238 10.1002/smll.201101791

[163] Santos M Tuzlakoglu K Fuchs S , et al. Endothelial cell colonization and angiogenic potential of combined nano-and micro-fibrous scaffolds for bone tissue engineering. Biomaterials 2008; 29: 4306–4313. 18706689 10.1016/j.biomaterials.2008.07.033

[164] Jiang Q Reddy N Yang Y . Cytocompatible cross-linking of electrospun zein fibers for the development of water-stable tissue engineering scaffolds. Acta Biomater 2010; 6: 4042–4051. 20438870 10.1016/j.actbio.2010.04.024

[165] Li H Cheng B Gao W , et al. Recent research progress and advanced applications of silica/polymer nanocomposites. Nanotechnol Rev 2022; 11: 2928–2964.

[166] Vasita R Katti DS . Nanofibers and their applications in tissue engineering. Int J Nanomed 2006; 1: 15–30. 10.2147/nano.2006.1.1.15 PMC2426767 17722259

[167] Subramanian A Krishnan UM Sethuraman S . Fabrication of uniaxially aligned 3D electrospun scaffolds for neural regeneration. Biomed Mater 2011; 6: 025004. 21301055 10.1088/1748-6041/6/2/025004

[168] Teo WE He W Ramakrishna S . Electrospun scaffold tailored for tissue-specific extracellular matrix. Biotechnol J 2006; 1: 918–929. 16941439 10.1002/biot.200600044

[169] Ma H Hu J Ma PX . Polymer scaffolds for small-diameter vascular tissue engineering. Adv Funct Mater 2010; 20: 2833–2841. 24501590 10.1002/adfm.201000922 PMC3911792

[170] Chehroudi B Gould TR Brunette DM . The role of connective tissue in inhibiting epithelial downgrowth on titanium-coated percutaneous implants. J Biomed Mater Res 1992; 26: 493–515. 1601902 10.1002/jbm.820260407

[171] Washburn NR Yamada KM Simon CG Jr. , et al. High-throughput investigation of osteoblast response to polymer crystallinity: influence of nanometer-scale roughness on proliferation. Biomaterials 2004; 25: 1215–1224. 14643595 10.1016/j.biomaterials.2003.08.043

[172] Kunzler TP Drobek T Schuler M , et al. Systematic study of osteoblast and fibroblast response to roughness by means of surface-morphology gradients. Biomaterials 2007; 28: 2175–2182. 17275082 10.1016/j.biomaterials.2007.01.019

[173] Wei G Ma PX . Partially nanofibrous architecture of 3D tissue engineering scaffolds. Biomaterials 2009; 30: 6426–6434. 19699518 10.1016/j.biomaterials.2009.08.012 PMC2763581

[174] Davis KA Burke KA Mather PT , et al. Dynamic cell behavior on shape memory polymer substrates. Biomaterials 2011; 32: 2285–2293. 21224032 10.1016/j.biomaterials.2010.12.006

[175] Thevenot P Nair A Dey J , et al. Method to analyze three-dimensional cell distribution and infiltration in degradable scaffolds. Tissue Eng C Methods 2008; 14: 319–331. 10.1089/ten.tec.2008.0221 PMC2913783 19055358

